# Coral anthozoan-specific opsins employ a novel chloride counterion for spectral tuning

**DOI:** 10.7554/eLife.105451

**Published:** 2025-09-01

**Authors:** Yusuke Sakai, Saumik Sen, Tomohiro Sugihara, Yukiya Kakeyama, Makoto Iwasaki, Gebhard FX Schertler, Xavier Deupi, Mitsumasa Koyanagi, Akihisa Terakita

**Affiliations:** 1 Department of Biology, Graduate School of Science, Osaka Metropolitan University, Sumiyoshi ku Osaka Japan; 2 Swiss Institute of Bioinformatics (SIB) Lausanne Switzerland; 3 Condensed Matter Theory Group, Laboratory for Theoretical and Computational Physics, PSI Center for Scientific Computing, Theory, and Data Villigen PSI Switzerland; 4 Laboratory of Biomolecular Research, PSI Center for Life Sciences Villigen PSI Switzerland; 5 The OMU Advanced Research Institute for Natural Science and Technology, Osaka Metropolitan University, Sumiyoshi-ku Osaka Japan; Leipzig University Germany; New York University United States

**Keywords:** coral, opsin, counterion, spectroscopy, GPCR, Other

## Abstract

Animal opsins are G protein-coupled receptors that have evolved to sense light by covalently binding a retinal chromophore via a protonated (positively charged) Schiff base. A negatively charged amino acid in the opsin, acting as a counterion, stabilizes the proton on the Schiff base, which is essential for sensitivity to visible light. In this study, we investigate the spectroscopic properties of a unique class of opsins from a reef-building coral belonging to the anthozoan-specific opsin II group (ASO-II opsins), which intriguingly lack a counterion residue at any of established sites. Our findings reveal that, unlike other known animal opsins, the protonated state of the Schiff base in visible light-sensitive ASO-II opsins is highly dependent on exogenously supplied chloride ions (Cl^−^). By using structural modeling and quantum mechanics/molecular mechanics (QM/MM) calculations to interpret spectroscopy data, we conclude that, in the dark state, ASO-II opsins employ environmental Cl^−^ as their native counterion, while a nearby polar residue, Glu292 in its protonated neutral form, facilitates Cl^−^ binding. In contrast, Glu292 plays a crucial role in maintaining the protonation state of the Schiff base in the light-activated protein, serving as the counterion in the photoproduct. Furthermore, Glu292 is involved in G protein activation of the ASO-II opsin, suggesting that this novel counterion system coordinates multiple functional properties.

## Introduction

Animals sense light by using opsins, photosensitive proteins belonging to the large family of G protein-coupled receptors (GPCRs). These proteins have a seven-transmembrane helix structure and bind to a retinal chromophore to form a light-sensitive pigment. Opsins are present in the genomes of all eumetazoans (i.e. all animal lineages except sponges), and based on their phylogenetic relationships, they can be classified into eight groups with distinctive properties: vertebrate visual/nonvisual opsins, opn3/TMT opsins, invertebrate Go-coupled opsins, cnidarian Gs-coupled opsins (cnidopsins), neuropsins (opn5), Gq-coupled visual pigments/melanopsins (opn4), peropsins, and retinochrome/RGR (Koyanagi and Terakita, 2014). Such diversity possibly underlies the diversification of light-dependent physiologies in animals. Furthermore, this diversity also provides a range of potential optogenetic tools to manipulate intracellular G protein-mediated signaling (Koyanagi and Terakita, 2014).

 Reef-building corals and sea anemones belong to the subphylum Anthozoa, which together with the subphylum Medusozoa constitute the phylum Cnidaria. Cnidarian animals possess multiple opsins categorized as part of the cnidarian Gs-coupled opsin group (cnidopsins), which regulate light-dependent processes (Koyanagi et al., 2008). For example, a member of this group is expressed in the ciliary-type visual cells of the box jellyfish lens eyes (Koyanagi et al., 2008; Kozmik et al., 2008). Beyond these Gs-coupled cnidopsins, anthozoan animals have opsins that are phylogenetically distinct from the other known eight groups and are found exclusively in anthozoans (Feuda et al., 2012; Suga et al., 2008). These anthozoan-specific opsins (ASO) can be further classified into two groups, ASO-I and ASO-II (Gornik et al., 2021; Hering and Mayer, 2014; Mason et al., 2023; Picciani et al., 2018; Ramirez et al., 2016). Gornik et al., 2021, proposed that both ASO-I and ASO-II were present in the last common ancestor of Anthozoa and Medusozoa but were lost secondarily in the Medusozoa lineage (Gornik et al., 2021). While it has been reported that both ASO-I and ASO-II are expressed in multiple tissues of sea anemones (Gornik et al., 2021; Suga et al., 2008) and corals (Levy et al., 2021), there is still a limited understanding of their molecular characteristics and physiological functions.

  The members of the ASO-II group are not only phylogenetically unique but also display interesting features in their amino acid sequences. For instance, several of these opsins lack an amino acid residue conserved among typical opsins that is crucial for absorption of visible light (Gornik et al., 2021; Mason et al., 2023). While free retinal in solution has its absorption maximum (λ_max_) in the ultraviolet (UV), this shifts to visible light when retinal is bound to a lysine residue in the transmembrane bundle of the opsin (usually at Lys296, numbering according to the bovine rhodopsin sequence) through a protonated Schiff base to form the pigment. Such a protonated Schiff base is necessary to achieve sensitivity to visible light in opsin-based pigments (Pitt et al., 1955). However, the proton on the positively charged Schiff base is energetically unstable in the hydrophobic transmembrane environment. To stabilize this proton, a negatively charged residue, glutamic or aspartic acid, is situated near the Schiff base to act as a counterion. This counterion is essential for opsin-based pigments to absorb visible light, and the residues serving as the counterion are highly conserved across opsins (Nathans, 1990; Terakita, 2005; Terakita et al., 2012). To date, three experimentally confirmed sites for the counterion have been identified in animal opsins: 94 in helix 2 (Gerrard et al., 2018), 113 in helix 3 (Nathans, 1990; Sakai et al., 2022; Sakmar et al., 1989; Zhukovsky and Oprian, 1989), and 181 in extracellular loop 2 (Nagata et al., 2019; Terakita et al., 2004; Terakita et al., 2000). Remarkably, some opsins belonging to the ASO-II group lack glutamic or aspartic acid at any of these established counterion positions (Gornik et al., 2021; Mason et al., 2023). This absence raises the question of whether these opsins can absorb visible light, and if so, by what mechanism.

  In this study, we investigate the spectroscopic properties of opsins in the ASO-II group isolated from the reef-building coral *Acropora tenuis*. Absorption spectra reveal that this group includes opsins sensitive to both UV and visible light. We then focus on a particular visible light-sensitive opsin within the ASO-II group (Antho2a) by spectroscopically analyzing the protonated and deprotonated states of the Schiff base in the wild-type and in single-point mutants. By interpreting the spectroscopy data in the light of hybrid quantum mechanics/molecular mechanics (QM/MM) simulations, we demonstrate that a chloride anion (Cl^−^) serves as a counterion to the retinylidene Schiff base in animal opsins, specifically in visible light-sensitive opsins of the ASO-II group.

## Results

### Identification of *A. tenuis* opsins

**Figure 1.**
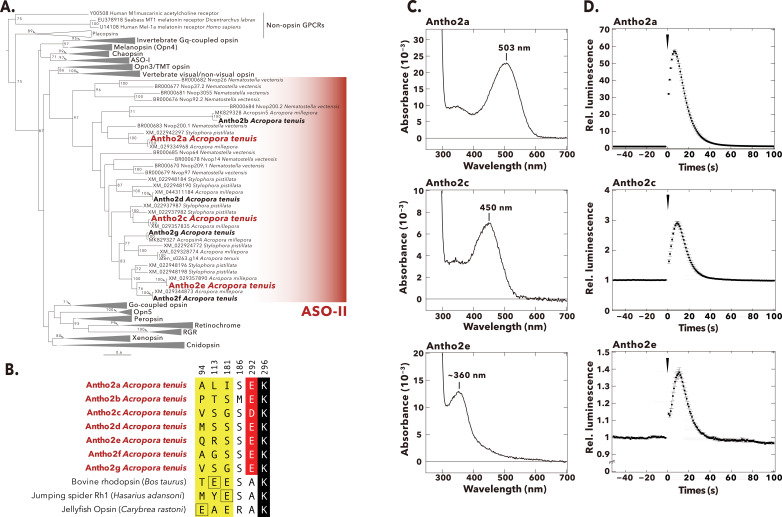
Phylogenetic tree, selected amino acid residues, absorption spectra, and light-induced Ca^2+^ responses of *A. tenuis* opsins belonging to the anthozoan-specific opsin II (ASO-II) group. (**A**) Maximum-likelihood (ML) tree of animal opsins including *A. tenuis* opsins in the ASO-II group. Seven opsins in the ASO-II group that were identified and cloned from *A. tenuis* in this study are shown in bold, and the three members for which we obtained absorption spectra are highlighted in red. Numbers at the nodes represent support values of each ML branch estimated by 1000 bootstrap samplings (≥70% are indicated). Scale bar = 0.6 substitutions per site. All branches and support values are provided in Figure 1—figure supplement 1. (**B**) Selected residues near the Schiff base in opsins of the ASO-II group and other animal opsins. Animal opsins typically have an acidic residue acting as counterion at one of three established sites (yellow): E94 (e.g. jellyfish opsin), E113 (e.g. bovine rhodopsin), or E181 (e.g. jumping spider Rh1). Remarkably, opsins in the ASO-II group lack an acidic residue at any of these positions but instead feature an acidic residue at position 292 (red). The retinal-binding lysine, Lys296, is shown in black. A more detailed sequence alignment is provided in Figure 1—figure supplement 2. Residues are numbered according to bovine rhodopsin. (**C**) Absorption spectra in the dark of three *A. tenuis* opsins in the ASO-II group (Antho2a, Antho2c, and Antho2e). The absorption spectra were measured at 0°C in 140 mM NaCl at pH 6.5. The number in each graph shows the λ_max_ value. (**D**) Results of the aequorin-based bioluminescent reporter assay for monitoring light-induced changes in Ca^2+^ in HEK293S cells expressing the same three opsins in the ASO-II group as in panel C. In each graph, luminescence values were normalized to the baseline. Black circles with error bars indicate the means ± SEMs (n=3) of the measured relative luminescence. Black arrowheads at time 0 indicate the timing of 1 min irradiation with green (495 nm; for Antho2a and Antho2c) or ultraviolet (UV) (395 nm; for Antho2e) light. Figure 1—source data 1.Raw absorbance values of purified pigments of Antho2a, Antho2c, and Antho2e recorded in the dark in the wavelength range of 250–750 nm. Figure 1—source data 2.Relative Ca^2+^ responses values (fold changes in luminescence above baseline levels) of wild types of Antho2a, Antho2c, and Antho2e.

We identified 17 opsins from the *A. tenuis* genome and transcriptome datasets by homology search, which included eight opsins in Gs-coupled cnidopsin group, one opsin in the ASO-I group, and eight opsins in the ASO-II group (Figure 1A; Figure 1—figure supplement 1). Full-length cDNAs of seven out of the eight opsins in the ASO-II group were isolated and cloned from adult or larval tissues of the coral (highlighted by bold letters in Figure 1A). We failed to amplify one opsin in the ASO-II group (gene model ID in the OIST Marine Genomics Unit Genome Project; Shinzato et al., 2021: aten_s0263.g14) by RT-PCR possibly because of its little mRNA expression level. Amino acid sequence alignment shows that all the seven *A. tenuis* opsins in the ASO-II group lack a glutamic or aspartic acid at the established counterion positions 94, 113, or 181 (Figure 1B; Figure 1—figure supplement 2). These opsins also have no E(D)RY motif at the cytoplasmic end of helix 3 (Figure 1—figure supplement 2), which is conserved throughout most class A GPCRs (Hofmann et al., 2009).

**Figure 1—figure supplement 1.**
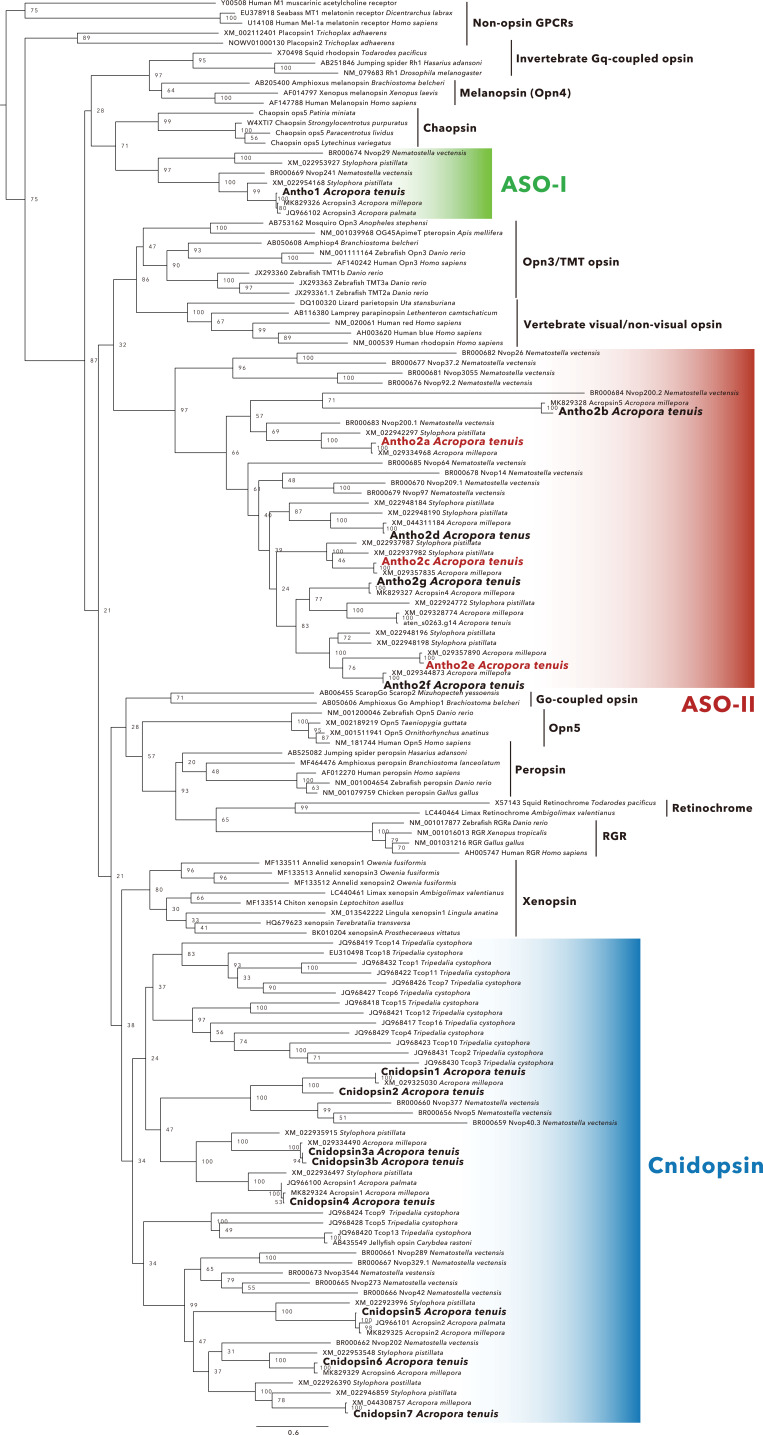
Maximum-likelihood (ML) tree of animal opsins, with non-opsin G protein-coupled receptors (GPCRs) included as an outgroup (a simplified version of the ML tree is shown in Figure 1A). The tree includes opsins belonging to the eight main groups (see main text) as well as opsins from the recently identified subgroups xenopsins and chaopsins (Ramirez MD et al. *Genome Biol Evol* **8**:3640–3652, 2016). The sixteen *A. tenuis* opsins (eight opsins in the cnidopsin group, one opsin in the anthozoan-specific opsin I [ASO-I] group, and seven opsins in the ASO-II group) that were identified in this study are shown in bold. The three opsins in the ASO-II group for which absorption spectra were successfully obtained are highlighted in red. Numbers at nodes represent support values for the ML branch estimated by 1000 bootstrap samplings (≥70% are indicated). Scale bar = 0.6 substitutions per site.

**Figure 1—figure supplement 2.**
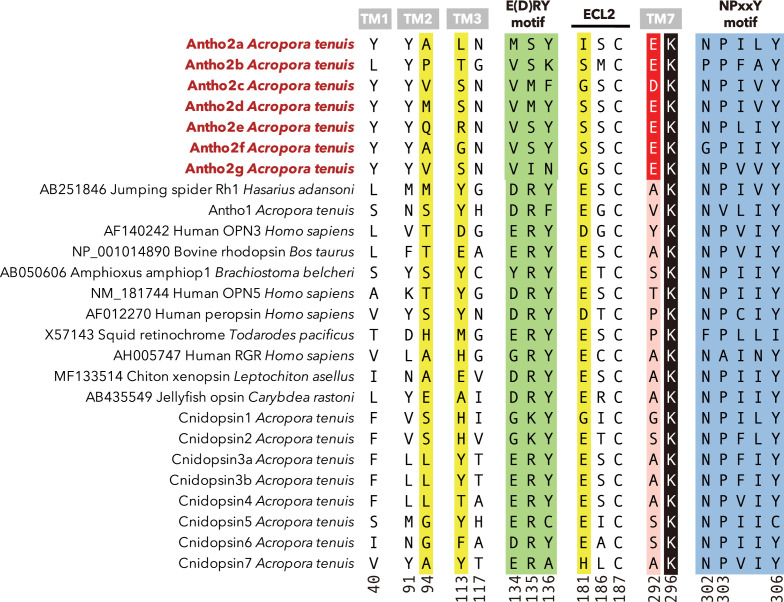
Key residues in opsins of the anthozoan-specific opsin II (ASO-II) group and other animal opsins. Residues near the Schiff base were selected with reference to the crystal structure of bovine rhodopsin (PDB ID: 1U19) and the homology model of Antho2a (shown in Figure 4). Position 292 contains an acidic residue (D/E) only in opsins of the ASO-II group (red). In addition, functionally important residues (such as the retinal binding Lys296 [black], the three established counterion sites [yellow], and two highly conserved motifs in Class A G protein-coupled receptors [GPCRs], E(D)RY on TM3 [green] and NPxxY on TM7 [blue]) are also shown. Residues are numbered according to bovine rhodopsin.

### Absorption spectra of *A. tenuis* opsins in the ASO-II group

We expressed seven members of the ASO-II group in COS-1 cells and purified their recombinant pigments in detergent-solubilized conditions. We successfully obtained the absorption spectra of three (Antho2a, Antho2c, and Antho2e) out of the seven members, which showed that Antho2a and Antho2c are visible light-sensitive opsins with λ_max_ at 503 nm and 450 nm, respectively, whereas Antho2e is a UV-sensitive opsin with λ_max_ at ~360 nm (Figure 1C). We have previously reported that one opsin in the ASO-II group, acropsin 4 of the coral *Acropora millepora*, induces a light-dependent elevation of intracellular Ca^2+^ levels (Mason et al., 2023). Here, we showed that Antho2a, Antho2c, and Antho2e evoked a similar light-dependent increase of Ca^2+^ levels in HEK293S cells (Figure 1D).

### Search for the counterion in *A. tenuis* opsins of the ASO-II group

Antho2a and Antho2c form visible light-sensitive pigments in the dark (Figure 1C) despite the lack of a negatively charged counterion at any of the established positions (Figure 1B; Figure 1—figure supplement 2). To investigate how the protonated Schiff base is stabilized in these opsins, we studied in more detail Antho2a (λ_max_ = 503 nm), as it could be expressed well in cultured cells and was stable in detergent-solubilized conditions (Figure 1C).

#### Contribution of Glu292 to the absorption spectra of the dark state and photoproduct of Antho2a

**Figure 2.**
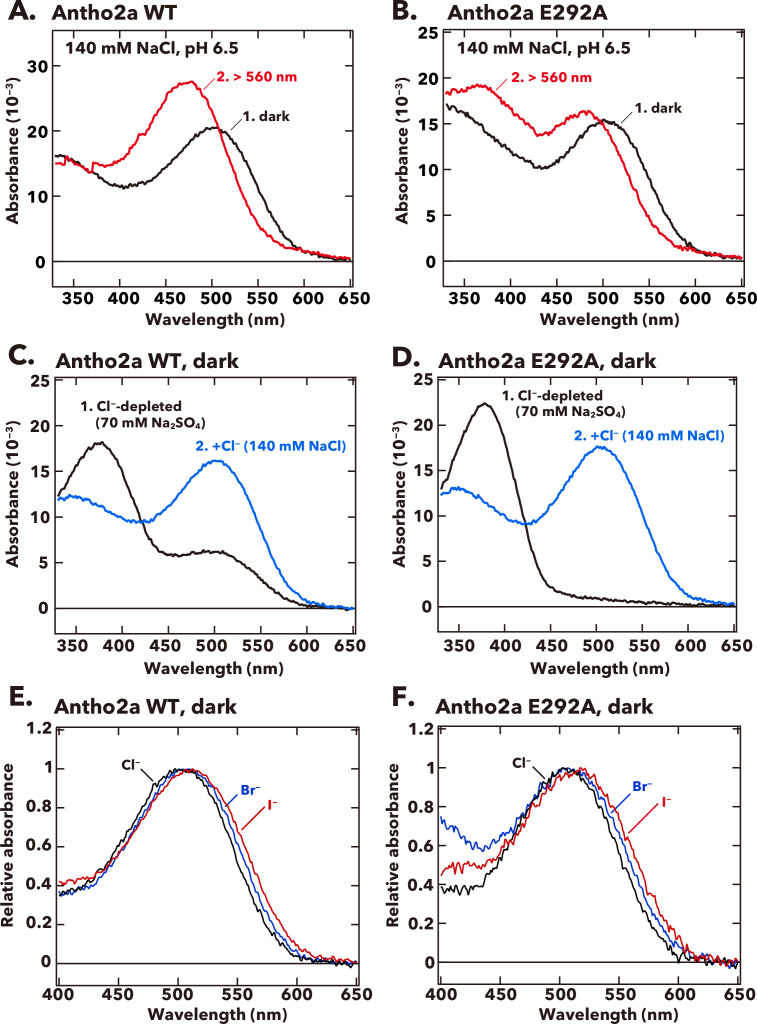
Absorption spectra of wild-type and the E292A mutant of *A. tenuis* Antho2a. (**A, B**) Absorption spectra of the dark state (curve 1, black) and the photoproduct (curve 2, red) of the wild-type (Antho2a WT, **A**) and the E292A mutant (Antho2a E292A, **B**) at 140 mM NaCl and pH 6.5. The samples were kept at 0°C during the spectroscopic measurements. (**C, D**) Absorption spectra of the dark state of Antho2a WT (**C**) and Antho2a E292A (**D**) prepared in Cl^−^-depleted conditions, before (curve 1, black) and after (curve 2, blue) adding Cl^−^ (see Materials and methods for details). In the Cl^−^-depleted condition, the pigments were solubilized in 70 mM Na_2_SO_4_, which reportedly does not access to the Cl^−^ binding site in the chicken red-sensitive cone visual pigment iodosin (Shichida et al., 1990) to moderate protein denaturation. (**E, F**) Effect of halide anions on the absorption spectra of wild-type Antho2a (**E**) and the Antho2a E292A mutant (**F**) at pH 6.5 and 0°C. The graphic shows the normalized absorption spectra of the pigments prepared in 140 mM NaCl (black curves), 140 mM NaBr (blue curves), and 140 mM NaI (red curves). Figure 2—source data 1.Raw absorbance values.

First, we searched for potential counterions at positions different from known established amino acid sites (91, 113, and 181) in the Antho2a sequence. Using the crystal structure of bovine rhodopsin (PDB ID: 1U19) as a template, we identified glutamic or aspartic acids located within 5 Å of the Schiff base in Antho2a and other members in the ASO-II group. Notably, all *A. tenuis* opsins in this group contain a conserved glutamic/aspartic acid at position 292 (Figure 1B; Figure 1—figure supplement 2), positioned just one helix turn away from the retinal-binding residue Lys296. To determine whether Glu292 could function as the counterion in Antho2a, we mutated Glu292 to alanine and measured the absorption spectra. The absorption spectrum of the E292A mutant in the dark was nearly identical to that of wild type (Figure 2A and B, curve 1), exhibiting a clear absorbance in the visible light region with only a slightly red-shifted λ_max_ (505 nm) at 140 mM NaCl and pH 6.5. This shows that a negative charge other than Glu292 may serve as a counterion in the dark state of wild-type Antho2a.

**Figure 2—figure supplement 1.**
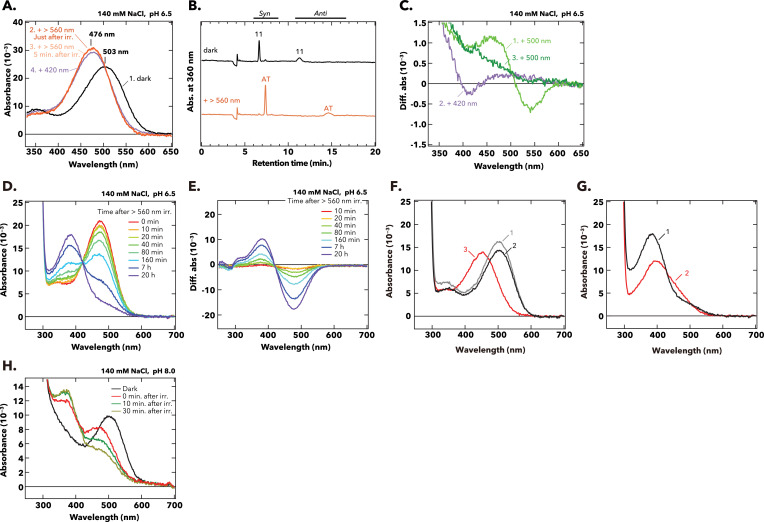
Photo- and thermal reactions of wild-type Antho2a. (**A**) Absorption spectra of purified wild-type Antho2a were measured at 0°C in the dark (curve 1, black), after orange light irradiation (>560 nm, curve 2 and curve 3, deep and pale orange), and after subsequent violet light irradiation (420 nm, curve 4, purple). Irradiation of wild-type Antho2a with orange light shifted the absorption maximum from 503 nm in the dark (curve 1, black) to 476 nm in the photoproduct (curve 2 and curve 3, orange). Upon subsequent irradiation with 420 nm light (while the photoproduct was stable), the λ_max_ of the photoproduct stayed at 476 nm, with only a slight decrease of the peak absorbance (curve 4, purple) possibly resulting from degradation of the photoproducts upon light irradiation. (**B**) The configuration of retinal in wild-type Antho2a before (black) and after (orange) irradiation with orange light (>560 nm) was analyzed with HPLC. Retinal was extracted in its oxime form. AT, all-*trans* retinal; 11, 11-*cis* retinal. (**C**) Difference spectra calculated from the absorption spectra recorded before and after sequential irradiations with 500 nm and 420 nm light for crude extracts of detergent-solubilized cell membranes expressing wild-type Antho2a. Curve 1: Difference spectrum of after minus before 500 nm irradiation, showing a blue-shifted spectral change indicative of photoproduct formation. Curves 2 and 3: Difference spectra of after minus before subsequent 420 nm light irradiation (curve 2), and after minus before a second 500 nm light irradiation, following the 420 nm irradiation (curve 3). Neither condition resulted in spectral changes consistent with regeneration of the dark state, indicating that Antho2a is not bistable under these conditions. (**D**) Changes in the absorption spectra of the purified pigment of wild-type Antho2a after irradiation with orange (>560 nm) light (with the sample kept in the dark at 0°C). Each colored curve corresponds to a different incubation time after the light irradiation. (**E**) Difference spectra obtained by subtracting the spectrum of wild-type Antho2a in the dark from the spectra measured at different time points after irradiation (shown in D). (**F, G**) Acid denaturation of pigments before (dark state, **F**) and after light irradiation (**G**). (**F**) Absorption spectra of wild-type Antho2a in the dark were measured immediately after sample preparation at pH 6.5 (curve 1), and after incubation overnight at 0°C (curve 2, pH 6.5) with subsequent addition of HCl to pH 1.9 (curve 3). (**G**) Absorption spectra of wild-type Antho2a incubated for 20 hr at 0°C following irradiation with orange light (>560 nm), measured at pH 6.5 (curve 1) and after acidification to pH 1.9 (curve 2). When retinal binds to opsin via a Schiff base (protonated or deprotonated), acid denaturation traps the chromophore as a protonated Schiff base, exhibiting absorption at λ_max_ ~440 nm. Acid denaturation of irradiated Antho2a (incubated for 20 hr post-irradiation) did not yield a product with λ_max_ at ~440 nm (G, curve 2), whereas the dark state pigment after acidification displayed absorption at ~440 nm (F, curve 3). These results indicate that Antho2a gradually releases retinal after light irradiation. (**H**) Absorption spectra of purified wild-type Antho2a at pH 8.0, measured at 0°C before (dark state; curve 1, black) and 0, 10, and 30 min after irradiation with orange light (>560 nm; red, green, and yellow curves, respectively). Figure 2—figure supplement 1—source data 1.Raw absorbance values and raw HPLC data.

**Figure 2—figure supplement 2.**
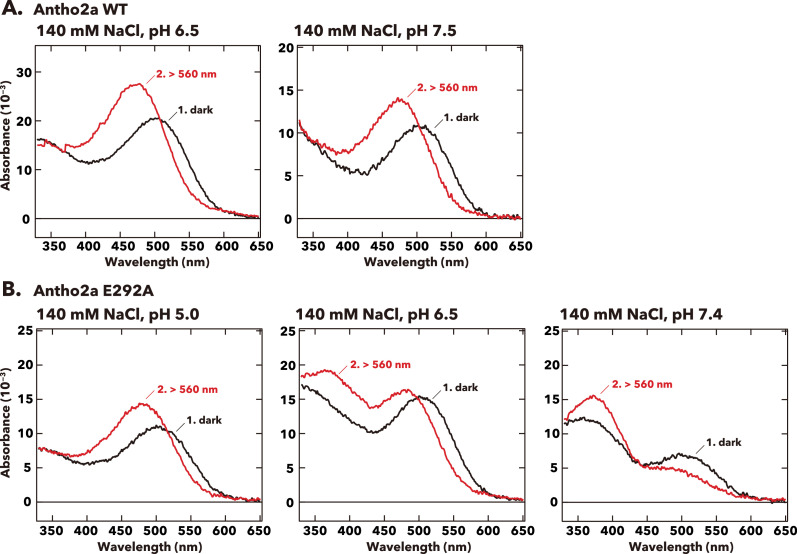
Effect of pH on the absorption spectra of the dark states and photoproducts of Antho2a. Absorption spectra of the dark state and photoproduct of (**A**) wild-type Antho2a and (**B**) the E292A mutant were measured at various pH conditions with keeping the NaCl concentration at 140 mM. Each graph shows spectra before (curves 1, black) and after (curves 2, red) irradiation with orange (>560 nm) light. Figure 2—figure supplement 2—source data 1.Raw absorbance values.

**Figure 2—figure supplement 3.**
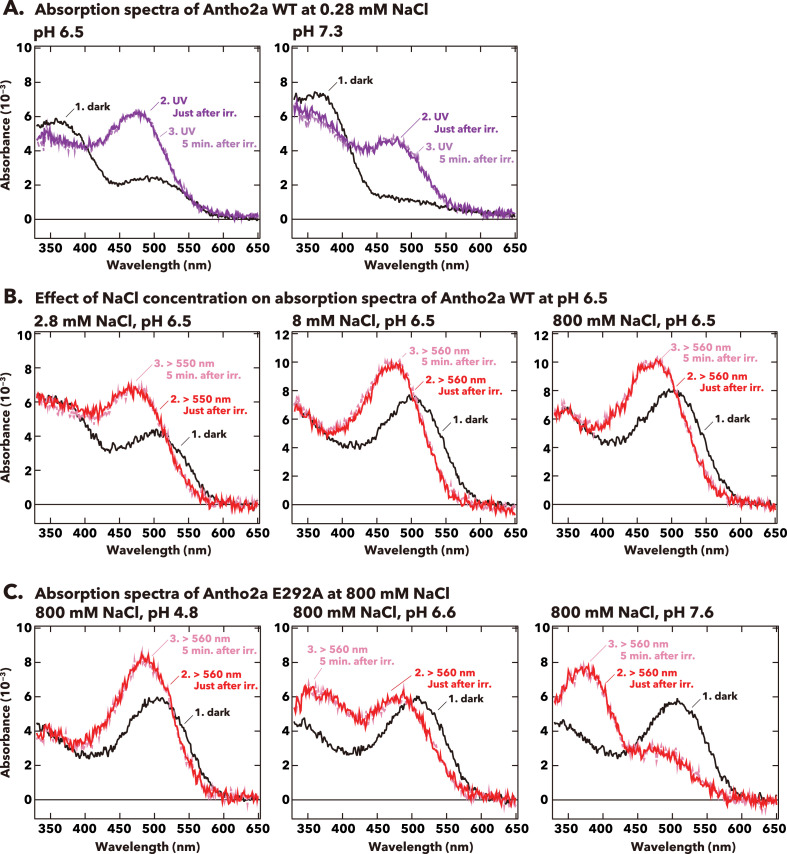
Effect of pH and NaCl concentration on absorption spectra of the dark states and photoproducts of Antho2a. (**A**) Absorption spectra of wild-type Antho2a at 0.28 mM NaCl under different pH conditions (pH 6.5 and pH 7.3). (**B**) Absorption spectra of wild-type Antho2a at pH 6.5 with varying NaCl concentrations (2.8 mM, 8 mM, and 800 mM). (**C**) Absorption spectra of the E292A mutant of Antho2a at 800 mM NaCl and different pH conditions (pH 4.8, pH 6.6, and pH 7.6). Each graph shows spectra before (curves 1, black) and after (curves 2, deep purple/red, and 3, pale purple/pink) irradiation with UV light (<410 nm, **A**) or orange light (>550 nm or >560 nm, **B and C**). Curves 2 and 3 in each graph represent, respectively, the first measurement (immediately after irradiation) and a subsequent measurement (<5 min after irradiation). The minimal change in the absorption spectra over this timescale indicates that the spectra remain stable after irradiation. Figure 2—figure supplement 3—source data 1.Raw absorbance values.

 We next investigated the spectroscopic properties of the photoproduct of wild-type Antho2a and the E292A mutant. Upon irradiation of wild-type Antho2a with orange light, the λ_max_ shifted from 503 nm in the dark to 476 nm in the photoproduct (Figure 2A, curve 2; Figure 2—figure supplement 1A, curves 2 and 3). This shift is due to the photoisomerization of the 11-*cis* retinal chromophore to its all-*trans* form, converting almost 100% of the dark state to the photoproduct (Figure 2—figure supplement 1B). The photoproduct remained stable for at least 5 min (Figure 2—figure supplement 1A, curves 2 and 3) but did not revert to the original dark state upon subsequent irradiation (Figure 2—figure supplement 1A and C). Instead, it underwent gradual decay accompanied by retinal release over time (Figure 2—figure supplement 1D–G). These findings indicate that purified Antho2a is neither strictly bleach resistant nor bistable (see also Figure 2—figure supplement 1 legend). We also observed that the protonated photoproduct decayed more rapidly at pH 8.0 (Figure 2—figure supplement 1H) than at pH 6.5 (Figure 2—figure supplement 1A, D, and E). In contrast to the dark state, the photoproduct of the E292A mutant displayed two distinct absorption peaks in UV and visible light regions, at ~370 nm and 476 nm, respectively (Figure 2B, curve 2). This suggests that the E292A mutation causes UV-light absorption due to a deprotonated Schiff base in the photoproduct. Additionally, altering the pH modified the ratio of absorbance between the ~370 nm and 476 nm peaks in the E292A mutant (Figure 2—figure supplement 2B, curves 2), with the UV-peak to the visible light-peak ratio increasing at higher pH levels (pH 7.4, Figure 2—figure supplement 2B, curve 2). Conversely, the wild type did not exhibit an increase in UV absorbance under similar high pH conditions (pH 7.5, Figure 2—figure supplement 2A, curve 2). These results indicate that the Schiff base in the photoproduct of the Antho2a E292A mutant has a lower acid dissociation constant (p*K*_a_) than that of the wild type, suggesting that Glu292 acts as the counterion in the photoproduct of Antho2a.

 We then further explored the nature of the counterion in the dark state of Antho2a. Previous studies have shown that in the bovine rhodopsin E113A and E113Q mutants, as well as in the retinochrome E181Q mutant (referred to as ‘counterion-less’ mutants), halide ions like Cl^−^ can act as ‘surrogate’ counterions to stabilize the proton on the Schiff base. Consequently, these counterion-less mutants can still absorb visible light in the presence of Cl^−^ (Nathans, 1990; Sakmar et al., 1991; Terakita et al., 2000). To assess the potential role of Cl^−^ as a surrogate counterion in the dark state of the Antho2a E292A mutant, we performed spectroscopic analyses under Cl^−^-depleted conditions (Figure 2C and D). We observed that the λ_max_ of the E292A mutant shifted to the UV region (Figure 2D, curve 1). Unexpectedly, a similar shift in absorption to the UV region was also observed in the wild type under the Cl^−^-depleted condition (Figure 2C, curve 1). These results indicate that, in the absence of Cl^−^, the Schiff base in both the wild-type and the E292A dark states becomes deprotonated. The subsequent addition of Cl^−^ (final concentration: 140 mM NaCl) restored clear absorbance in the visible light region (Figure 2C and D, curves 2), showing that Cl^−^ facilitates the protonation of the Schiff base of the dark state even in the wild type. In contrast, the photoproduct of the wild type exhibited no significant change in the ratio of UV to visible-light absorption peaks at pH 6.5 across NaCl concentrations from 0.28 mM to 800 mM (Figure 2—figure supplement 3). The photoproduct of the wild type consistently absorbed visible light under these NaCl conditions (curves 2 in Figure 2—figure supplement 3A and B), suggesting that Cl^−^ has little impact on the Schiff base p*K*_a_ in the photoproduct of wild-type Antho2a. However, the photoproduct of the E292A mutant exhibited a pH-dependent shift in the ratio of UV to visible-light absorption between pH 4.8 and pH 7.6, even at 800 mM NaCl, where the dark state predominantly absorbed visible light (Figure 2—figure supplement 3C). This further supports that Glu292 serves as the counterion in the photoproduct of Antho2a.

#### Effect of halide anions on λ_max_ values of the dark state of Antho2a

To obtain further evidence supporting the Cl^−^ counterion in the dark state of Antho2a, we examined the impact of different halide anions on the absorption spectrum in the dark state of Antho2a, as observed in the bovine rhodopsin counterion-less mutant (Nathans, 1990; Sakmar et al., 1991). Antho2a readily absorbed visible light in the presence of bromide ion (Br^−^) and iodide ion (I^−^), as well as Cl^−^, and the λ_max_ of wild-type Antho2a shifted depending on the halide solutions (503 nm in 140 mM NaCl; 506 nm in 140 mM NaBr; 511 nm in 140 mM NaI solutions; Figure 2E). The E292A mutant showed a similar shift in λ_max_ (505 nm in 140 mM NaCl; 507 nm in 140 mM NaBr; 517 nm in 140 mM NaI solutions; Figure 2F).

#### Effect of Cl^−^ concentration on the p*K*_a_ of the protonated Schiff base of Antho2a

**Figure 3.**
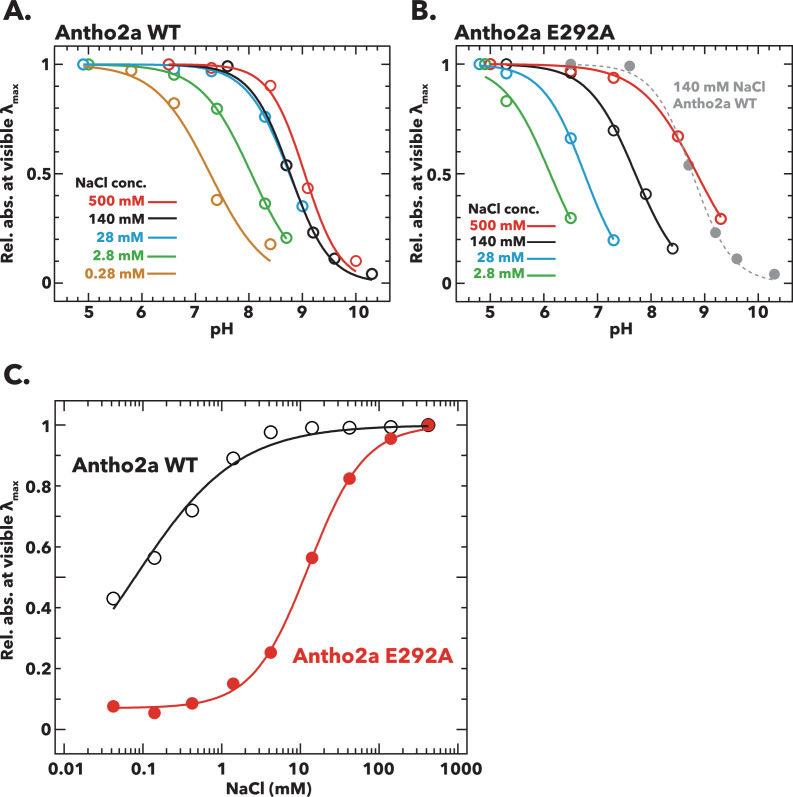
Effects of pH and Cl^−^ concentration on the absorption spectra of the dark states of wild-type Antho2a and the Antho2a E292A mutant. (**A, B**) Changes in the absorbance at λ_max_ as a function of pH for (**A**) wild-type Antho2a and (**B**) the E292A mutant at different Cl^−^ concentrations. The absorbance values at ‘visible λ_max_’ (mean absorbance at 503±5 nm for the wild type and 505±5 nm for the E292A mutant, respectively) were normalized for each Cl^−^ concentration to those at the lowest pH, in which the Schiff base is assumed to be fully protonated (‘Rel. abs. at visible λ_max_’ in the y-axes). Solid and dashed lines represent sigmoid fits to the experimental data for each Cl^−^ concentration (indicated by different colors). The pH-dependent change of wild-type Antho2a at 140 mM NaCl is also shown in panel B (dotted gray line). The full absorption spectra used to generate these plots are provided in Figure 3—figure supplement 1 (for wild-type Antho2a) and Figure 3—figure supplement 3 (for the E292A mutant). (**C**) Changes in the absorbance at λ_max_ for wild-type Antho2a (black open circles) and the E292A mutant (red solid circles) as a function of Cl^−^ concentration. The absorbance values at visible λ_max_ were normalized to those at 500 mM NaCl for both the wild type and the E292A mutant. The lines in the graph were generated by fitting the Hill equation to the experimental data. The full absorption spectra used to generate these plots are provided in Figure 3—figure supplement 4. Figure 3—source data 1.Summary of mean relative absorbance values at λ_max_ (±5 nm) at different pH and NaCl concentrations.

To further investigate the influence of Cl^−^ on the protonation state of the Schiff base in the dark state of Antho2a, we estimated the p*K*_a_ of the Schiff base by measuring the pH-dependent changes in the absorption spectra of Antho2a at different Cl^−^ concentrations. The pH-dependent equilibrium between the visible (protonated Schiff base) and UV (deprotonated Schiff base) forms revealed that their ratio changes with Cl^−^ concentration (Figure 3—figure supplement 1A–E). A plot of the changes in absorbance at λ_max_ against pH (Figure 3A) shows that in wild-type Antho2a, the p*K*_a_ of the protonated Schiff base increases with higher Cl^−^ concentrations (7.3 at 0.28 mM NaCl, 8.0 at 2.8 mM, 8.8 at 28 mM, 8.8 at 140 mM, and 9.0 at 500 mM). We failed to determine the p*K*_a_ at 0 mM NaCl, as the observed λ_max_ in acidic conditions (pH <6.5) was shorter than expected in Antho2a (503 nm), suggesting that a normal pigment was not produced under these conditions (Figure 3—figure supplement 2A). Similarly, the Cl^−^ concentration also affected the p*K*_a_ of the protonated Schiff base in the E292A mutant (6.1 at 2.8 mM NaCl, 6.8 at 28 mM, 7.7 at 140 mM, and 8.9 at 500 mM) (Figure 3B; Figure 3—figure supplement 3). At 0 mM NaCl, the E292A mutant showed no visible light absorption, even under the most acidic conditions (pH 4.7), preventing the determination of its p*K*_a_ (Figure 3—figure supplement 2B). Notably, at low Cl^−^ concentrations (2.8 mM NaCl), the wild type exhibited a higher p*K*_a_ than the E292A mutant (8.0 and 6.1, respectively). However, at 500 mM NaCl, the p*K*_a_ of the E292A mutant and wild type were comparable (9.0 and 8.9, respectively; Figure 3A and B). These results suggest that Cl^−^, rather than Glu292, serves as the counterion in the dark state of Antho2a, while Glu292 facilitates the protonation of the Schiff base by the Cl^−^ counterion.

**Figure 3—figure supplement 1.**
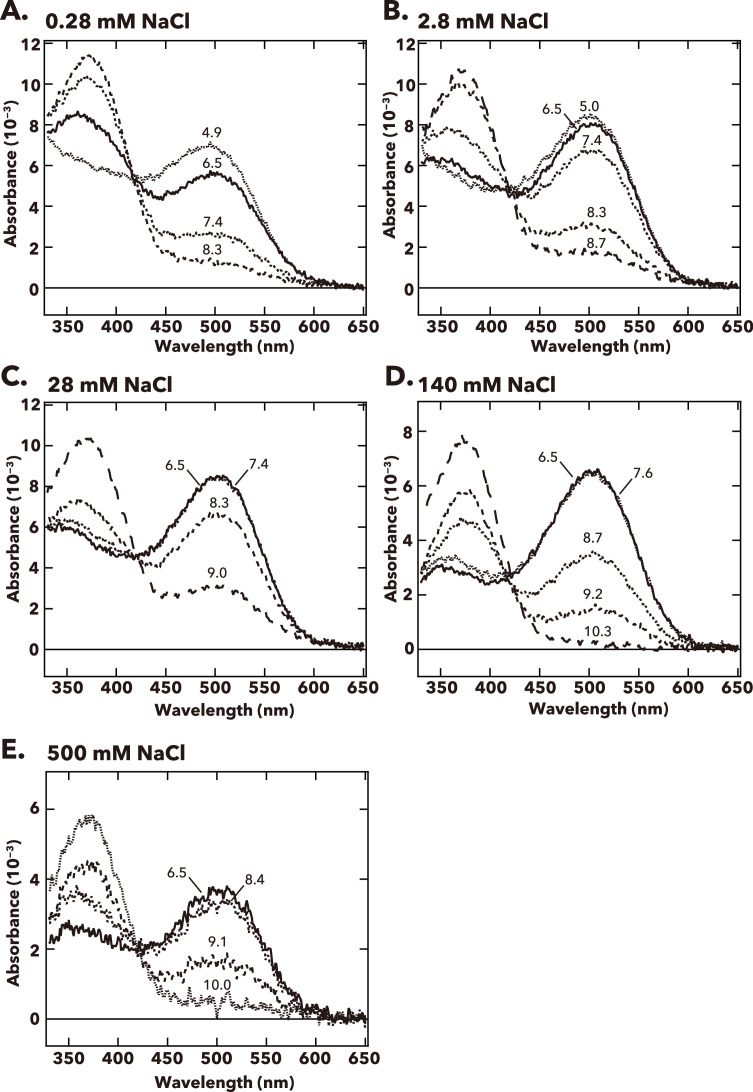
pH-dependent changes in the absorption spectra of Antho2a wild type (WT) at (**A**) 0.28 mM, (**B**) 2.8 mM, (**C**) 28 mM, (**D**) 140 mM, and (**E**) 500 mM Cl^−^ at 0°C. The pH values of the solution, measured right after each spectroscopic measurement, are indicated on the corresponding curves in each graph. Figure 3—figure supplement 1—source data 1.Raw absorbance values.

**Figure 3—figure supplement 2.**
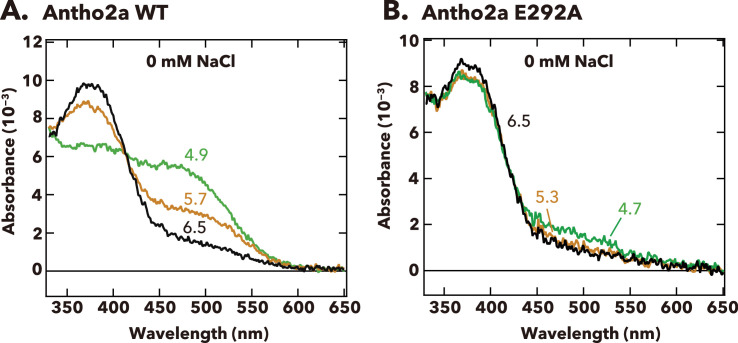
pH-dependent changes in the absorption spectra of Antho2a wild type (WT) and Antho2a E292A at 0 mM NaCl (containing 70 mM Na_2_SO_4_) at 0°C. The pH values at which the absorption spectra were measured are indicated on the corresponding curves. Figure 3—figure supplement 2—source data 1.Raw absorbance values.

**Figure 3—figure supplement 3.**
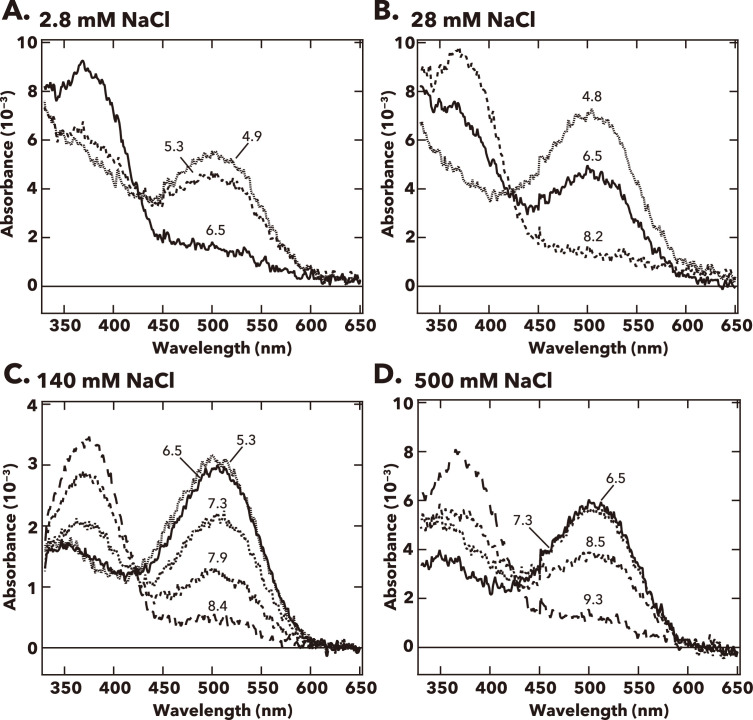
pH-dependent changes in the absorption spectra of Antho2a E292A at (**A**) 2.8 mM, (**B**) 28 mM, (**C**) 140 mM, and (**D**) 500 mM Cl^−^ at 0°C. The pH values are indicated on the corresponding curves in each graph. Figure 3—figure supplement 3—source data 1.Raw absorbance values.

**Figure 3—figure supplement 4.**
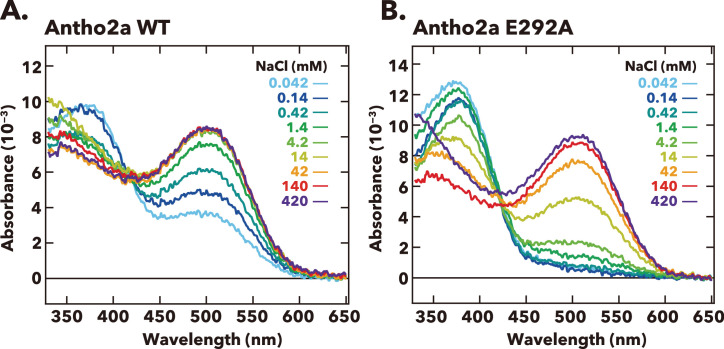
Absorption spectra of (**A**) wild-type Antho2a and (**B**) the Antho2a E292A mutant under different Cl^−^ concentrations at pH 6.5 and 0°C. Each color indicates a different concentration of Cl^−^. Figure 3—figure supplement 4—source data 1.Raw absorbance values.

#### Binding affinity of Cl^−^ to wild-type Antho2a and the E292A mutant

To evaluate the Cl^−^ binding affinities of both wild-type Antho2a and the E292A mutant, we measured changes in their absorption spectra by gradually increasing Cl^−^ concentrations at pH 6.5 and estimated the Cl^−^ dissociation coefficients (K_d_). The relative absorbance in the visible region increased with higher Cl^−^ concentrations both in the wild type and in the E292A mutant (Figure 3—figure supplement 4A and B). By fitting the Hill equation to the experimental data (Figure 3C), the dissociation constants (K_d_) of Cl^−^ were determined to be 0.079±0.010 mM for the wild-type Antho2a and 12.7±0.519 mM for the E292A mutant. This significant increase in the K_d_ value for the E292A mutant suggests that the Cl^−^ binding affinity is considerably reduced due to the mutation. Consequently, we suggest that while Glu292 does not act as a direct counterion, it plays a crucial role in facilitating Cl^−^ binding to Antho2a.

### Structural modeling and QM/MM calculations of the dark state of Antho2a

**Figure 4.**
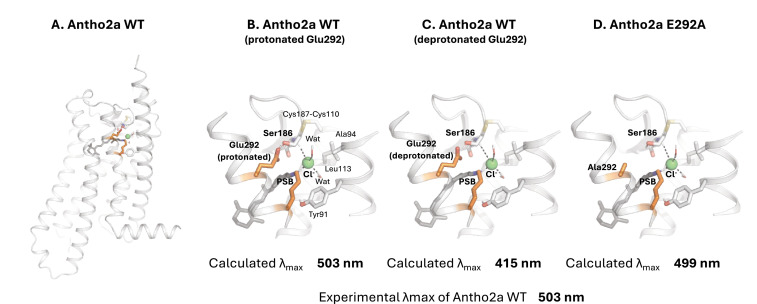
Quantum mechanics/molecular mechanics (QM/MM) structural model of wild-type Antho2a in the dark state (**A**) and detailed views of the retinal binding pocket with a protonated (neutral) Glu292 (**B**), a deprotonated (negatively charged) Glu292 (**C**), and the E292A mutant (**D**). The retinal protonated Schiff base (PSB) and the binding pocket residues are shown as sticks (including polar hydrogens) and the Cl^−^ ion as a sphere with its coordination shown as dashes. ‘Wat’ indicates a water molecule. Residues in the QM region are marked in bold.

To gain a deeper understanding of the environment surrounding the retinylidene Schiff base in the dark state of Antho2a, we performed QM/MM-based structural modeling of both the wild-type Antho2a (with Glu292 either neutral or negatively charged) and the E292A mutant. The QM/MM geometry optimization positioned the Cl^−^ ion close to the Schiff base (~3 Å) and near Glu292 (~4.7 Å), with Glu292 itself located in proximity to the Schiff base (~3.3 Å) (Figure 4B). The chloride ion is also coordinated by two water molecules and the backbone of Cys187 which is part of a conserved disulfide bridge (Figure 1—figure supplement 2). The retinylidene Schiff base region also includes polar (Ser186, Tyr91) and nonpolar (Ala94, Leu113) residues (Figure 4). To validate these models, we calculated the QM/MM vertical excitation energies of the ground state geometries (Table 1).

**Table 1.** Vertical excitation energies (Δ*E_calc_*) and oscillator strengths (*f*) computed by quantum mechanics/molecular mechanics (QM/MM) calculations using different QM methods with the cc-pVTZ basis set.

QM Region	sTD-DFT CAM-B3LYP		ADC(2)	
	Δ*E*_*calc*_ nm (eV)	*f*	Δ*E*_*calc*_ nm (eV)	*f*
RET +Lyr296+Cl + Ser186+**Glu292 (deprotonated)**	415 (2.99)	1.54	374 (3.32)	1.64
RET +Lyr296+Cl + Ser186+**Glu292 (protonated)**	503 (2.47)	1.19	416 (2.98)	1.43
RET +Lyr296+Cl + Ser186+**Ala292**	499 (2.49)	1.19	426 (2.91)	1.36

 For wild-type Antho2a with a protonated neutral Glu292, the calculated λ_max_ using the CAM-B3LYP/cc-pVTZ level of theory was 503 nm (Figure 4B), in good agreement with the experimentally observed value (503 nm; Figure 2A). In contrast, the λ_max_ calculated with a deprotonated negatively charged Glu292 was blue-shifted to 415 nm (Figure 4C), deviating significantly from the experimental value. Finally, the calculated λ_max_ for the E292A mutant was 499 nm (Figure 4D), also in agreement with the experimental value (505 nm). To further substantiate these findings, we recalculated the excitation energies using the RI-ADC(2)/cc-pVTZ method. Although these λ_max_ values are blue-shifted compared to those calculated with the CAM-B3LYP method, they followed a similar trend. Both of these computational methods have previously been employed to accurately calculate the excitation energies of rhodopsins (Church et al., 2021). These results strongly suggest that in the dark state of Antho2a, Glu292 is protonated and neutral at pH 6.5, and therefore, it does not function as the counterion.

### Effect of the Glu292 mutation on the function of the photoproduct

**Figure 5.**
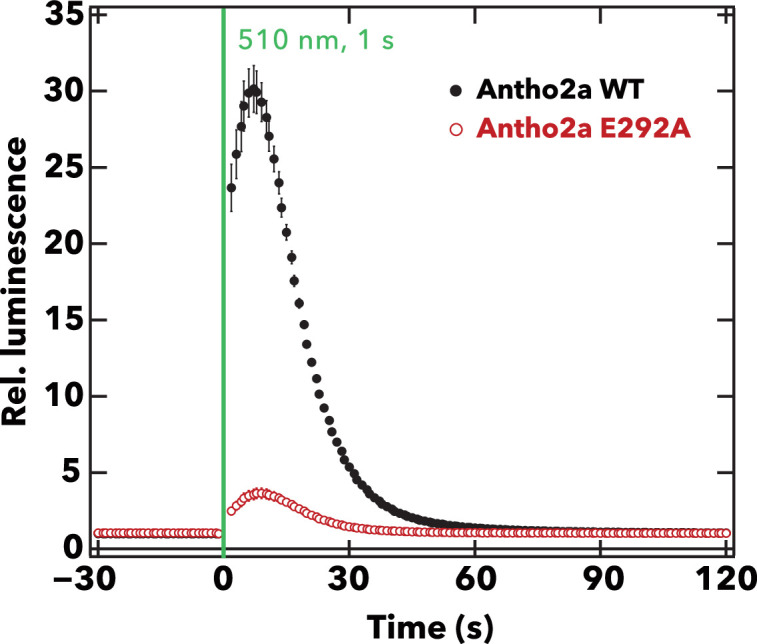
Comparison of the light-evoked intracellular Ca^2+^ levels between wild-type Antho2a and the E292A mutant. The graph shows the mean ± SEM (n=4) of the measured relative luminescence values (luminescence values normalized to the baseline) for wild-type Antho2a (black) and the E292A mutant (red) at pH 7.0. The green vertical line indicates the time of cell illumination with green light (510 nm, for 1 s, 1.65×10^15^ photons/cm^2^/s). Figure 5—source data 1.Mean and SEM values of relative Ca2+ 1024 responses (fold changes in luminescence above baseline levels) of wild type and E292A Antho2a.

The spectroscopy data indicate that Glu292 is involved in stabilizing the protonated Schiff base by facilitating Cl**^−^** binding in the dark state and also serves as a counterion in the photoproduct. This suggests that Glu292 significantly contributes to the visible light absorption of Antho2a. To explore additional roles of Glu292 in Antho2a, we measured the light-induced Ca^2+^ response in cultured cells expressing wild-type Antho2a or the E292A mutant. Notably, cells expressing wild-type Antho2a showed an ~30-fold increase in Ca^2+^ levels upon light irradiation (Figure 5, solid black circles), whereas cells expressing the Antho2a E292A mutant showed a smaller Ca^2+^ elevation (<5-fold increase) (Figure 5, red open circles). This indicates that the peak Ca^2+^ response in cells expressing wild-type Antho2a was approximately nine times greater than in cells expressing the E292A mutant. This result, along with the crucial role of Glu292 in Cl**^−^** binding in the dark state and as a counterion in the photoproduct, suggests that Glu292 also plays a role in G protein activation.

**Figure 5—figure supplement 1.**
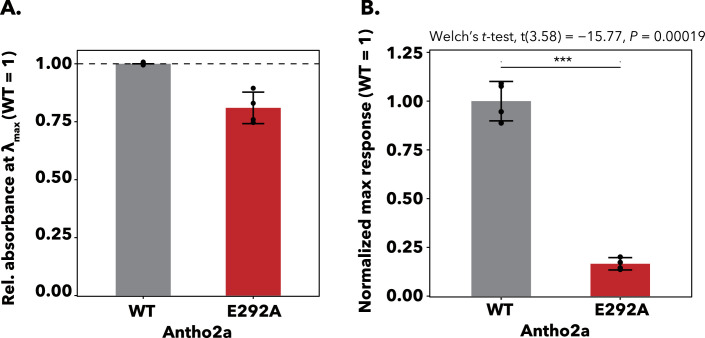
Expression levels and normalized Ca^2+^ responses of wild-type and E292A Antho2a. (**A**) The relative absorbances at λ_max_ (± 5 nm) of wild-type (gray) and E292A (red) Antho2a are used as indicators of expression levels. The proteins were expressed and purified under identical conditions, and absorbance values of the E292A mutant were normalized to those of the wild type. Spectra were measured at 0°C, pH 6.5, and 140 mM NaCl. Each bar represents mean ± SEM of n = 4 replicates from separate transfections in cultured cells. (**B**) Normalized maximum Ca^2+^ responses of HEK293S cells expressing wild-type Antho2a and the E292A mutant after irradiation with green light (510 nm). Responses were normalized to the expression levels shown in panel** A**. Each bar represents mean ± SEM of n = 4 replicates from separate transfections in cultured cells. Statistical evaluation of the normalized Ca^2+^ responses for wild-type Antho2a versus the E292A mutant was conducted using a Welch’s t-test (two-sided). ***p<0.001. Figure 5—figure supplement 1—source data 1.Summary of relative expression values and normalized Ca^2+^ response values for wild type and E292A mutant of Antho2a. Figure 5—figure supplement 1—source code 1.R code for analyzing Ca^2+^ response data in Figure 5—figure supplement 1—source data 1.

### Cl^−^-dependent changes in the absorption spectra of the dark states of Antho2c and Antho2e

**Figure 6.**
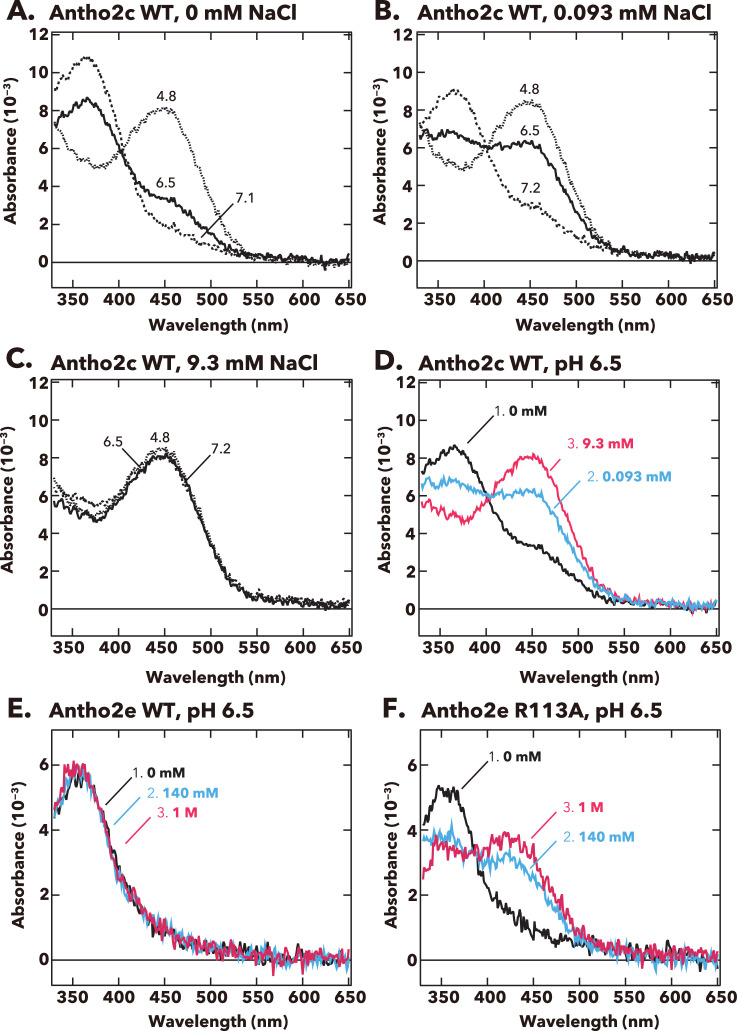
pH-dependent changes in the absorption spectra of Antho2c and Antho2e at different Cl^−^ concentrations at 0°C. (**A–C**) Absorption spectra of purified wild-type Antho2c pigment at (**A**) 0 mM, (**B**) 0.093 mM, and (**C**) 9.3 mM NaCl concentrations. The corresponding pH values are indicated on each curve in the graphs. (**D**) Summary of the spectral changes for wild-type Antho2c across different Cl^−^ concentrations at neutral pH (pH 6.5). (**E, F**) Absorption spectra of (**E**) wild-type Antho2e (Antho2e WT) and (**F**) its R113A mutant (Antho2e R113A) at different Cl^−^ concentrations at pH 6.5 at 0°C. Each color indicates a different Cl^−^ concentration. Figure 6—source data 1.Raw absorbance values.

We tested whether Cl^−^ concentration affects the p*K*_a_ of the Schiff base in another visible light-sensitive opsin, Antho2c (λ_max_ = 450 nm, Figure 1C). The pH-dependent equilibrium between UV- and visible-light absorbing forms was clearly observed at 0 mM or 0.093 mM NaCl, but not at 9.3 mM NaCl, where Antho2c stably absorbed visible light across the measured pH range (pH 4.8–7.2, Figure 6A–C). Also, the ratio of UV to visible-light absorption increased with higher Cl^−^ concentrations at pH 6.5 (Figure 6D). These results demonstrate that Cl^−^ serves as a counterion in the dark state of Antho2c, as it does in Antho2a. In contrast, wild-type Antho2e continues to absorb UV light even at 1 M NaCl (Figure 6E). Notably, Antho2e has an arginine at position 113, which corresponds to the counterion position in vertebrate visual opsins (Figure 1—figure supplement 2). When this arginine is mutated to alanine (R113A), the mutant becomes sensitive to visible light (λ_max_ = ~420 nm) in the presence of Cl^−^ (Figure 6F), suggesting that Cl^−^ can serve as the counterion in the R113A mutant.

## Discussion

**Figure 7.**
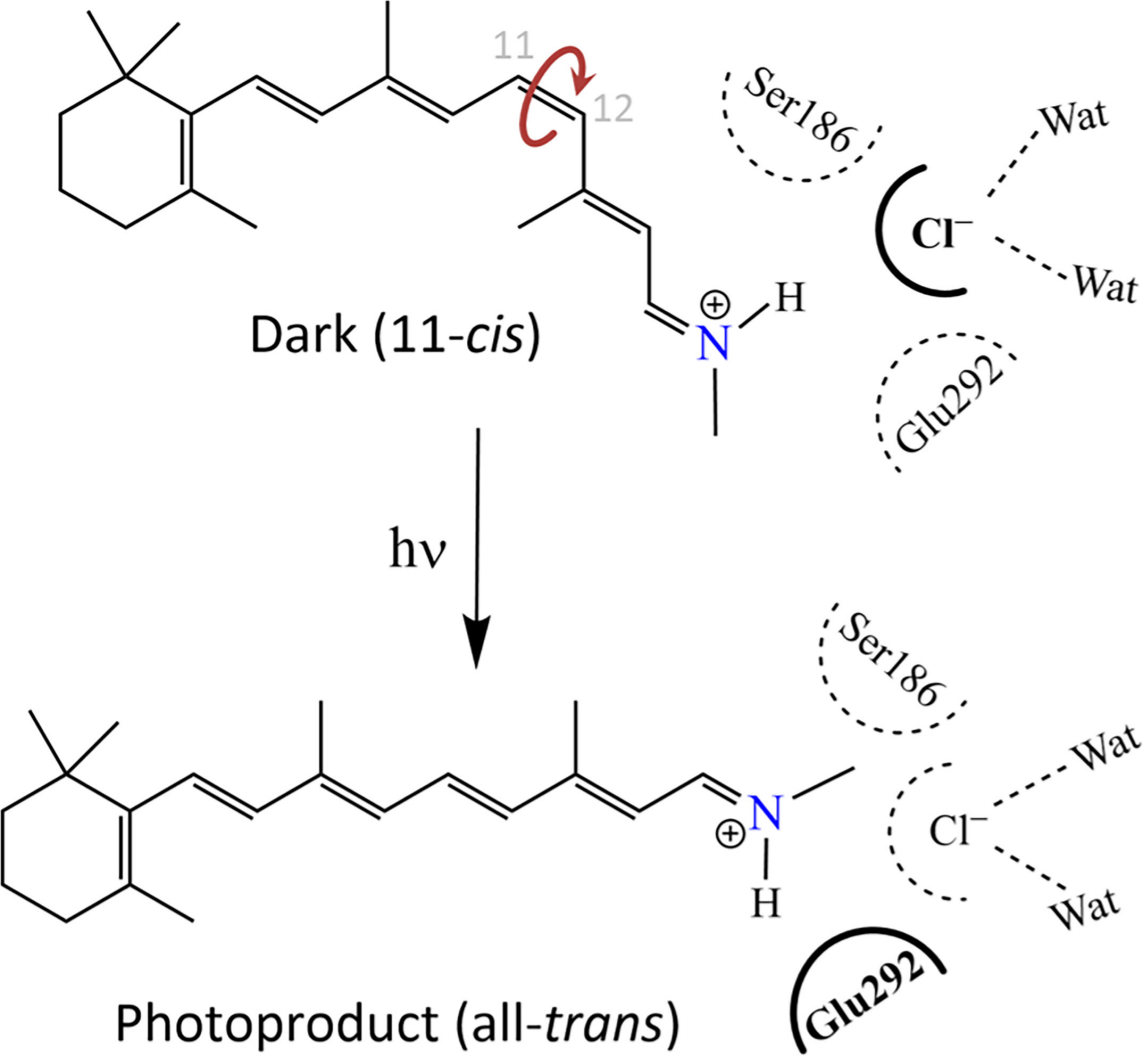
Schematic drawing of the environment of the protonated Schiff base depicting the counterion switch from a chloride ion (top) to Glu292 (bottom) upon light activation.

In this study, we reveal for the first time the spectral properties of opsins in the ASO-II group from the coral *A. tenuis*, showing that their sensitivity spans from UV to visible light. Opsins in this group have a highly conserved Glu292 residue near the Schiff base, which can potentially stabilize the proton on the Schiff base. Indeed, our results show that the p*K*_a_ of the protonated Schiff base in the photoproduct (λ_max_ = 476 nm) of Antho2a is altered when Glu292 is substituted with alanine (Figure 2A and B; Figure 2—figure supplement 2), suggesting that Glu292 serves as the counterion of the photoproduct. Conversely, the dark state of Antho2a (λ_max_ = 503 nm) exhibits robust visible light absorption only in the presence of Cl^−^ at physiological pH, and the p*K*_a_ of the protonated Schiff base changes with Cl^−^ concentration in both wild-type Antho2a and the E292A mutant. Furthermore, the p*K*_a_ for wild type and the E292A mutant is comparable in the presence of sufficient Cl^−^ (500 mM NaCl, Figure 3A and B), supporting the conclusion that Cl^−^, and not Glu292, acts as the counterion of the dark state of *A. tenuis* Antho2a. We found that the type of halide anions in the solution has a small but noticeable effect on the λ_max_ values of the dark state of Antho2a. This is consistent with the effect observed in a counterion-less mutant of bovine rhodopsin, in which halide ions serve as surrogate counterions (Nathans, 1990; Sakmar et al., 1991). Similarly, our results align with earlier observations that the λ_max_ of a retinylidene Schiff base in solution increases with the ionic radius of halides acting as hydrogen bond acceptors (i.e. I^−^>Br^−^>Cl^−^) (Blatz et al., 1972). In contrast, the λ_max_ of halorhodopsin from *Natronobacterium pharaonic* does not clearly correlate with halide ionic radius (Scharf and Engelhard, 1994), as the halide ion in this case is not a hydrogen-bonding acceptor of the protonated Schiff base (Kouyama et al., 2010; Mizuno et al., 2018). Altogether, these findings support our hypothesis that in Antho2a, a solute halide ion forms a hydrogen bond with the Schiff base, thereby serving as the counterion in the dark state. Moreover, QM/MM calculations for the dark state of Antho2a suggest that Glu292 is protonated and neutral, further supporting the hypothesis that Glu292 does not serve as the counterion in the dark state. However, unlike the dark state, Cl^−^ has little to no effect on the visible light absorption of the photoproduct (Figure 2—figure supplement 3). Therefore, we conclude that Cl^−^ and Glu292, respectively, act as counterions for the protonated Schiff base of the dark state and photoproduct of Antho2a. This represents a unique example of counterion switching from exogenous anion to a specific amino acid residue upon light irradiation (Figure 7).

 Our spectroscopic data also showed that the other visual light-sensitive opsin, Antho2c, exhibited the Cl^−^ dependency on the Schiff base p*K*_a_ of the dark state, which suggested that opsins in the ASO-II group may share a spectral tuning mechanism based on the Cl^−^ counterion. Interestingly, Antho2e had an arginine at position 113, and when it was mutated to alanine (R113A), the mutant showed sensitivity to visible light in the presence of Cl^−^ (Figure 6F). We hypothesize that the positive charge of Arg113 disturbs interaction between the Cl^−^ and the protonated Schiff base or that it completely inhibits Cl^−^ binding, rendering Antho2e UV-sensitive.

 It is noteworthy that although Cl^−^ has been reported to serve as a surrogate counterion in ‘counterion-less’ mutants of animal opsins (such as E113Q in bovine rhodopsin and E181Q in retinochrome) (Nathans, 1990; Sakmar et al., 1991; Terakita et al., 2000), Antho2a is, to our knowledge, the first example in a wild-type animal opsin that employs Cl^−^ as a counterion. Interestingly, within the microbial rhodopsin family, heliorhodopsin (TaHeR) incorporates a Cl⁻ into the Schiff base region under high Cl⁻ concentrations at pH 4.5. This Cl⁻ stabilizes the protonated retinal Schiff base when its primary counterion, E108, is neutralized (Besaw et al., 2022). The observed 18 nm red shift at low pH is consistent with E108 protonation. The TaHeR E108A mutant shows the same λ_max_ under high Cl⁻ concentrations, further supporting the role of Cl⁻ as a counterion. At pH 8, the Schiff base proton is stabilized by the negatively charged E108 (Shihoya et al., 2019).

 The E292A mutation in Antho2a drastically decreases the Cl^−^ binding affinity in the dark state at pH 6.5 (K_d_: Antho2a WT = 0.079 mM, Antho2a E292A=12.7 mM). Based on these results, supported by our QM/MM calculations of the dark state, we hypothesize that protonated Glu292 not only serves as a counterion of the photoproduct but also constitutes part of the Cl^−^ binding site in the dark state of Antho2a. In Cl^−^-pumping microbial rhodopsins, where Cl^−^ serves as a counterion, the Cl^−^ binding site near the Schiff base is typically formed by hydrogen-bonding network between Cl^−^, the protonated Schiff base, and key amino acids such as serine, threonine, and glutamic/aspartic acid, and water molecules (Besaw et al., 2020; Hosaka et al., 2016; Kolbe et al., 2000). Additionally, the bovine rhodopsin double mutant E113Q/A292E exhibits a higher sensitivity to hydroxylamine than the wild type, indicating instability of the Schiff base and increased solvent accessibility (Tsutsui and Shichida, 2010). These observations suggest that Glu292 in Antho2a may also facilitate the accessibility of Cl^−^ ions to the Schiff base environment. To determine the precise nature of the Cl^−^ binding site and clarify the roles of Glu292 and other residues in the Cl^−^-dependent counterion system of Antho2a, detailed spectroscopic and structural experiments will be necessary.

 We also found that cells expressing the Antho2a E292A mutant show a lower Ca^2+^ elevation upon light stimulation compared to the wild type (Figure 5). The relative expression level of the E292A mutant of Antho2a was approximately 0.81 of the wild type (set as 1), as determined by comparing absorbances at λ_max_ for both pigments expressed and purified under identical conditions. Additionally, the fraction of protonated pigment relative to the wild type (set as 1 at pH 6.5) was estimated to be 0.94 for the E292A mutant at pH 6.5, and 0.99 and 0.84 for the wild type and the E292A mutant at pH 7.0, respectively (Figure 3A and B). Since pH 7.0 corresponds to the conditions used in the live-cell Ca^2+^ assays, the effective amount of protonated pigment for the E292A mutant was approximately 73% of the wild type. Nevertheless, even after normalization for these differences, the Ca^2+^ response amplitude of the E292A mutant remained significantly lower (~17% of wild type, compared to the observed 12% prior to normalization; Figure 5, Figure 5—figure supplement 1). These observations suggest that Glu292 serves not only as a counterion in the photoproduct but also plays an allosteric role in influencing G protein activation. It has been reported that the introduction of Glu or Asp at position 292 can affect various molecular properties of opsins. For instance, the A292E mutation in human rhodopsin has been shown to result in constitutively active apoproteins, leading to congenital night blindness (Dryja et al., 1993; Jin et al., 2003). These data suggest that Glu292 has the potential to influence functional properties, such as G protein activation. Several studies have reported that the counterion can affect diverse properties of opsins beyond their absorption spectra. For instance, in bovine rhodopsin, Glu113 is involved not only in visible light absorption in the dark state but also in the efficient activation of G proteins by the photoproduct (Terakita et al., 2004). It has been suggested that these pleiotropic functions of the counterion have been achieved through evolutionary modifications of the protein structure once the counterion was acquired (Terakita et al., 2004). This concept may similarly apply to Antho2a.

 One of the notable features of the opsins in the ASO-II group that use Cl^−^ as a counterion is the relatively low p*K*_a_ of the Schiff base (e.g. ~9.0 for Antho2a in the dark state, Figure 3A) compared to other animal opsins with a ‘regular’ amino acid counterion (e.g. >16 for bovine rhodopsin, with Glu113 as a counterion [Steinberg et al., 1993] or 11 for jumping spider Rh1, with Glu181 as a counterion [Nagata et al., 2019]). We hypothesize that opsins in the ASO-II group, with lower Schiff base p*K*_a_, may change their spectral sensitivity and G protein activation profile within the physiological pH range of corals. For instance, these opsins could exhibit decreased sensitivity to visible and increased sensitivity to UV light under alkaline pH conditions. The extra- and intracellular pH environments in symbiotic cnidarians such as corals are spatially and temporally variable due to the photosynthesis of symbiotic algae (Barott et al., 2017). For example, pH values in the cytosol of algae-hosting cells (a group of endodermal cells with algal occupancy) in corals reportedly increase by approximately 0.5 pH units upon light treatment (Venn et al., 2009). The pH values in the gastrovascular cavity (coelenteron), which is in contact with endodermal cells, range from 6.6 to 8.5 (Agostini et al., 2012; Al-Horani et al., 2003; Cai et al., 2016) and increase in the presence of light, presumably due to the consumption of CO_2_ by photosynthesis (Barott et al., 2017). Additionally, alkalinization in the extracellular region of calcifying cells reportedly increases ~1.0 pH units from dark to light conditions, reaching levels above pH 9.0 (Al-Horani et al., 2003). These pH changes, generally an increase due to photosynthetic activity, may result in variable light sensitivity of the ‘low p*K*_a_’ opsins in the ASO-II group. Recent studies have reported that members of the ASO-II group may be associated with the symbiotic relationship between host anthozoans and symbiotic algae. For example, using the symbiotic sea anemone *Exaiptasia diaphana*, Gornik et al., 2021, showed that the mRNA levels of several opsins in the ASO-II group were higher in symbiotic adults than in apo-symbiotic ones (Gornik et al., 2021). In sea anemone, it has also been reported that behavioral responses to light differ between symbiotic and apo-symbiotic individuals (Foo et al., 2020; Kishimoto et al., 2023), with these responses potentially being driven by the activation of ASO-II opsins that are upregulated in the presence of symbiotic algae. Moreover, single-cell RNA-seq analysis in the reef-building coral *Stylophora pistillata* has revealed that an opsin belonging to the ASO-II group is specifically expressed in algae-hosting cells (a group of endodermal cells with 50% algal occupancy) (Levy et al., 2021). Although further studies are needed, we suggest that the unique use of Cl^−^ as the counterion in opsins of the ASO-II group, rather than a negatively charged amino acid, may be associated with their pH-sensitive light response and, ultimately, to their role in photosynthesis-related functions in symbiotic cnidarians.

 It is widely accepted that opsins have evolved from a non-opsin GPCR (Feuda et al., 2012). Our finding of the native chloride counterion in opsins shows a possibility that in the first stage of the evolutionary process, the ‘primitive’ opsins having Lys296 but no counterion residue could absorb not only UV light but also a wide range of wavelengths that extends into the visible region by embracing chloride ions. Namely, the chloride counterion system might have been a preliminary step in the evolution of amino acid counterions in animal opsins. Further empirical and bioinformatic studies are required for disentangling the evolutionary trajectories of the Schiff base-counterion system, including the chloride counterion.

## Materials and methods

**Key resources table:** 

Reagent type (species) or resource	Designation	Source or reference	Identifiers	Additional information
Gene (*Acropora tenuis*)	Antho2a	This study	GenBank: LC844932	The sequence information is available from NCBI GenBank.
Gene (*Acropora tenuis*)	Antho2c	This study	GenBank: LC844934	The sequence information is available from NCBI GenBank.
Gene (*Acropora tenuis*)	Antho2e	This study	GenBank: LC844936	The sequence information is available from NCBI GenBank.
Recombinant DNA reagent	pUSRα-Antho2a_1D4	This paper		The coding sequence (CDS) of Antho2a was tagged with rho 1D4 epitope and inserted into the multicloning site of pUSRα vector (see Materials and methods section). Available from Akihisa Terakita lab.
Recombinant DNA reagent	pMT-Antho2a_1D4	This study		The CDS of Antho2a was tagged with rho 1D4 epitope and inserted into the multicloning site of pMT vector (see Materials and methods section). Available from Akihisa Terakita lab.
Recombinant DNA reagent	pUSRα-Antho2c_1D4	This study		The CDS of Antho2c was tagged with rho 1D4 epitope and inserted into the multicloning site of pUSRα vector (see Materials and methods section). Available from Akihisa Terakita lab.
Recombinant DNA reagent	pMT-Antho2c_1D4	This study		The CDS of Antho2c was tagged with rho 1D4 epitope and inserted into the multicloning site of pMT vector (see Materials and methods section). Available from Akihisa Terakita lab.
Recombinant DNA reagent	pUSRα-Antho2e_1D4	This study		The CDS of Antho2e was tagged with rho 1D4 epitope and inserted into the multicloning site of pUSRα vector (see Materials and methods section). Available from Akihisa Terakita lab.
Recombinant DNA reagent	pMT-Antho2c_1D4	This study		The CDS of Antho2e was tagged with rho 1D4 epitope and inserted into the multicloning site of pMT vector (see Materials and methods section). Available from Akihisa Terakita lab.
Recombinant DNA reagent	pcDNA3.1+/mit-2mutAEQ	Addgene	RRID:Addgene_45539	
Cell line (African green monkey)	COS-1	David Farrens lab.	RRID:CVCL_0223	
Cell line (*Homo sapiens*)	Human embryonic kidney 293S (HEK293S)		RRID:CVCL_A784	
Commercial assay or kit	In-Fusion HD cloning kit	TAKARA	Cat no. 639650	
Chemical compound, drug	PEI MAX - Transfection Grade Linear Polyethyleneimine Hydrochloride	Kyfora Bio	24765	
Chemical compound, drug	Dodecyl β-D-maltoside	DOJIMBO	D316-12	
Software, algorithm	IGOR Pro 8	https://www.wavemetrics.com/		
Software, algorithm	MAFFT v7	Katoh and Standley, 2013		
Software, algorithm	ModelTest-NG v0.2.0	Darriba et al., 2020		
Software, algorithm	RAxML-NG v1.2.0	Kozlov et al., 2019		
Software, algorithm	AlphaFold2	Jumper et al., 2021		
Software, algorithm	HomolWat	Mayol et al., 2020		
Software, algorithm	PROPKA	Olsson et al., 2011		
Software, algorithm	AMBER	Case et al., 2025		
Software, algorithm	Orca 5.0.2	Neese, 2022		
Software, algorithm	ChemShell 3.7.1	Metz et al., 2014		
Software, algorithm	Turbomole 7.5.1	Furche et al., 2014		
Software, algorithm	PyMOL 2.5.5.	The PyMOL Molecular Graphics System, Version 2.5.5 Schrödinger, LLC		

### Experimental design

We first identified and cloned opsins from a reef-building coral, *A. tenuis*, and then expressed opsins belonging to the ASO-II group in mammalian cultured cells. We performed spectroscopic measurements of purified pigments of the opsins in different pH and Cl^−^ conditions to identify their effects on the acid dissociation constant of the protonated Schiff base of the opsins, leading to the determination of the counterion. Computational modeling and QM/MM calculations were also conducted to elucidate the retinylidene Schiff base environment in the dark state of Antho2a. Light-evoked Ca^2+^ responses were assessed by aequorin-based bioluminescent reporter assay to evaluate the G protein activation of Antho2a.

### Identification of *A. tenuis* opsins and phylogenetic tree inference

*Acropora tenuis* (Dana, 1846) is a common reef-building coral distributed throughout the Indo-Pacific Ocean. Candidate sequences of *A. tenuis* opsins were identified by homology search against public genome and transcriptome datasets (Shinzato et al., 2021; Voolstra et al., 2015), and their phylogenetic relationships to known opsins were inferred by subsequent phylogenetic tree reconstruction. We first conducted BLASTP and TBLASTN searches with an E-value cutoff of 10^–10^ using *Acropora palmata* Acropsin 1–3 (JQ966100-JQ966102), two *Nematostella vectensis* opsins (BR000676-BR000677), human rhodopsin (NM_000539), and squid rhodopsin (X70498) as queries. We aligned the collected opsin homologs and excluded sequences that did not contain a retinal-binding lysine residue (Lys296) in the seventh transmembrane helix. We modified the fragmented sequences by reference to the genome sequence of *A. tenuis* and opsin sequences of other *Acropora* species (*A. palmata* or *A. millepora*). The candidate sequences of *A. tenuis* opsins were combined with the representative opsin sequences. The final sequence set was aligned using MAFFT (Katoh and Standley, 2013) and trimmed by TrimAl (Capella-Gutiérrez et al., 2009) with the ‘*gappyout*’ function. The ML tree was reconstructed using RAxML-NG v1.1.0 (Kozlov et al., 2019) assuming the LG+G4 model of protein evolution, which was selected by ModelTest-NG v0.2.0 (Darriba et al., 2020). The ML branch supports were estimated with 1000 bootstrap replicates.

### Sample collection, total RNA extraction, and cDNA synthesis

Colonies of *A. tenuis* were collected from <3 m depth on the fringing reef on Sesoko Island, Okinawa (N26°37.58′, E127°52.01′) and were maintained in flow-through aquaria at Sesoko Station (Tropical Biosphere Research Center, University of Ryukyus, Okinawa, Japan). Four days after spawning, motile larvae and small branches of adult colonies were preserved in RNAlater Stabilization Solution (Thermo Fisher Scientific, MA, USA). Total RNAs were extracted from the larval and adult samples using TRIzol reagent (Thermo Fisher Scientific) or Sepasol-RNA I Super G (nacalai tesque, Kyoto, Japan) and purified using QIAGEN RNeasy Mini Kit (QIAGEN, Hilden, Germany) following the manufacturer’s protocol. cDNAs were synthesized from the total RNA by reverse transcription using High-Capacity cDNA Reverse Transcription kits (Thermo Fisher Scientific).

### Expression and purification of *A. tenuis* opsins

The coding regions of *A. tenuis* opsins were amplified by PCR with gene-specific primers and were tagged with the epitope sequence of the anti-bovine rhodopsin antibody rho 1D4 (ETSQVAPA) at their C-termini. Site-directed mutants were produced by overlap extension PCR using PrimeSTAR Max DNA Polymerase (TAKARA, Shiga, Japan) with site-specific primers and were also tagged with the 1D4 epitope sequence. The tagged cDNAs were inserted into the pUSRα vector (Kayada et al., 1995) digested with HindIII and EcoRI or the pMT vector (Ridge and Abdulaev, 2000) digested with EcoRI and NotI using In-Fusion HD cloning kit (TAKARA). The plasmids (15 µg per 100 mm culture dish) were transfected into COS-1 cells using the polyethyleneimine (PEI) transfection method as described previously (Obayashi et al., 2025; Sinha et al., 2014). The transfected cells were maintained for 24 hr after transfection at 37°C under 5% CO_2_ and then 11-*cis* retinal was added to the medium (1 µL of 4 mM 11-*cis* retinal to 100 mm culture dish) following 25°C or 30°C incubation for another 24 hr in the dark before collecting the cells. The reconstituted pigments were extracted from the cell membranes with 1% dodecyl β-D-maltoside (DDM, Dojindo, Kumamoto, Japan), 50 mM HEPES, and 140 mM NaCl (pH 6.5). The solubilized samples were mixed with 1D4-conjugated agarose beads overnight, and the mixture was transferred into Bio-Spin columns (Bio-Rad, Hercules, CA, USA) and washed in the buffer containing 0.02% DDM, 50 mM HEPES, and 140 mM NaCl (pH 6.5, buffer A). The purified pigments were eluted with buffer A containing 0.5–1 mg/mL 1D4 peptide (custom peptide synthesis by GenScript Japan Inc, Tokyo, Japan). To obtain pigments in solutions of various anions (SO_4_^2−^, Br^−^, and I^−^) other than Cl^−^, samples were prepared as described above and in the final step, the mixture of solubilized samples and 1D4-agarose beads was washed with buffer A followed by the additional wash with buffers including different sodium salts of anions (0.02% DDM, each of 70 mM Na_2_SO_4_, 140 mM NaBr, or 140 mM NaI, and 50 mM HEPES). Then, the pigments were eluted with the buffer including the appropriate sodium salt of anion containing 0.5–1 mg/mL 1D4 peptide. Alternatively, for some pigments that were unstable in the absence of Cl^−^, we quickly removed Cl^−^ by gel-filtration chromatography on PD MiniTrap desalting columns with Sephadex G-25 resin (Cytiva, Marlborough, MA, USA). The columns were first equilibrated with the buffer including 0.02% DDM, 70 mM Na_2_SO_4_, and 50 mM HEPES, 500 µL of samples were loaded onto the columns and eluted with the buffer. We collected 800 µL fractions and used them for subsequent spectroscopic analyses.

### UV-visible spectroscopy

Spectroscopic measurements were performed at 0°C using a V-750 UV-visible spectrophotometer (JASCO Corporation, Tokyo, Japan). The pH of the samples was adjusted with 100 mM CAPS, including NaOH for alkaline conditions and 500 mM NaH_2_PO_4_ for acidic conditions. pH values were measured using a pH meter (B-211; HORIBA, Kyoto, Japan) immediately after each spectroscopic measurement. The concentration of Cl^−^ in the samples was adjusted by addition of different concentrations of NaCl solutions which were prepared in 70 mM Na_2_SO_4_ buffer (see above). A 100 W halogen lamp was equipped on the spectrophotometer and used to illuminate samples with a set of optical interference filters (420 nm or 500 nm, Toshiba) and cutoff filters (O-55 or O-56, AGC Techno Glass Co., Shizuoka, Japan). Absorption spectra of some UV-absorbing pigments were recorded using the V-750 UV-visible spectrophotometer, equipped with a 300 W xenon lamp (MAX-350; Asahi Spectra Co., Tokyo, Japan) that was used for illumination of samples in combination with a UV-transmitting filter (UTVAF-50S-36U, SIGMA KOKI, Tokyo, Japan).

### HPLC analysis

An HPLC analysis was carried out to analyze the conformations of retinal present in the purified pigments as described previously (Terakita et al., 1989), with some modifications. Briefly, 100 µL of purified pigments were mixed with 210 µL of cold 90% methanol which was stored in −20°C and 30 µL of 1 M hydroxylamine to convert retinal chromophore in a sample into retinal oxime. The retinal oxime was extracted with 700 µL of *n*-hexane. 200 µL of the extract were injected into a YMC-Pack SIL column (particle size 3 μm, 150×6.0 mm^2^) and eluted with *n*-hexane containing 15% (vol/vol) ethyl acetate and 0.15% (vol/vol) ethanol at a flow rate of 1 mL/min.

### Bioluminescent reporter assays for Ca^2+^ measurements in cultured cells

Ca^2+^ levels in opsin-expressing cultured cells were assessed by an aequorin-based luminescent assay as described previously (Koyanagi et al., 2022). Briefly, the plasmid containing open reading frames of each opsin was transfected into HEK293S cells in 35 mm dishes by the PEI method with the aequorin plasmid obtained by introducing a reverse mutation A119D into the plasmid (pcDNA3.1+/mit-2mutAEQ) (Addgene no. 45539) (de la Fuente et al., 2012). The transfected HEK293S cells were incubated for ~24 hr at 37°C under 5% CO_2_ with the addition of 0.2 µM/dish of 11-*cis* retinal 4–5 hr after the transfection. Before the luminescence measurements, the culture medium was replaced with a medium containing coelenterazine h (pH 7.0), and the cells were incubated to equilibrate with the media at 25°C for at least 2 hr. Dishes of cells were then stimulated with light, and luminescence values were recorded using GloMax 20/20n Luminometer (Promega). A green (495 nm) LED light (color: ‘Cyan’, Ex-DHC; BioTools Inc, Gunma, Japan) and arrayed LEDs on a board with spectral emission peaks at 390 nm and 510 nm (SPL-25-CC; REVOX Inc, Kanagawa, Japan) were used as light sources.

### Cell lines

The identities of the HEK293S cell line used in the study were authenticated by short-tandem repeat profiling. The COS-1 cells were kindly provided by Dr. David Farres (Oregon Health & Science University) and have been maintained in the laboratory. We have checked that both HEK293S cell and COS-1 cell lines were free from mycoplasma contamination using real-time PCR.

### Computational modeling and QM/MM calculations

The three-dimensional structure of Antho2a was predicted from the primary amino acid sequence using AlphaFold2 (Jumper et al., 2021). The 11-*cis* retinal chromophore linked to the protonated Schiff base was incorporated into the AlphaFold model using as a template the high-resolution structure of bovine rhodopsin solved by time-resolved serial femtosecond X-ray crystallography (Gruhl et al., 2023). A Cl^−^ anion was initially placed in close proximity to the retinal protonated Schiff base, as observed in the microbial chloride-pump halorhodopsin (Mous et al., 2022). Water molecules were added using HomolWat (Mayol et al., 2020). We determined the p*K*_a_ values of the titratable amino acid residues at pH 6.5 using the PROPKA program (Olsson et al., 2011; Søndergaard et al., 2011) and subsequently, the protein was protonated using the tleap program in the AMBER software package (Case et al., 2016). The geometry of this initial model was first relaxed by molecular mechanics energy minimization with the Amber ff14SB force field (Maier et al., 2015) using steepest descent for 10,000 steps before switching to a conjugate gradient minimizer for an additional 10,000 steps. During energy minimization, a positional restraint of 10 kcal/mol/Å^2^ was applied to all atoms, including hydrogens. The SHAKE algorithm (Ryckaert et al., 1977) was used to constrain the motion of bonds involving hydrogen. Finally, the geometry of the system was optimized using hybrid QM/MM calculations without considering any external environment and with the backbone of the protein frozen (Mroginski et al., 2021; Senn and Thiel, 2009). The QM part consists of the retinal chromophore linked to the lysine side chain cut between the Cδ and Cε atoms forming the protonated Schiff base, along with Cl^−^, Glu292, and Ser186. The retinal-binding pocket also contains predicted water molecules (modeled based on homologous GPCR structures) close to the Schiff base and the chloride ion, which were not included in the QM region. The hydrogen link atom scheme was used at the QM/MM boundary. The QM part was treated using the BP86-D3 (BJ) functional (Becke, 1988; Grimme et al., 2011) in conjunction with the cc-pVDZ basis set (Dunning, 1989) and the def2/J auxiliary basis set for the resolution of identity (RI) (Weigend, 2008). The chain of spheres exchange algorithm was utilized in combination with the resolution of identity for the Coulomb term (RI-J). The rest of the protein was treated with the Amber ff14SB force field. Water molecules were treated with the TIP3P model (Jorgensen et al., 1983). The QM/MM calculations were performed using the quantum chemistry program Orca 5.0.2 (Neese, 2022) interfaced with the DL_POLY module of the ChemShell 3.7.1 software package (Metz et al., 2014; Sherwood et al., 2003). The optimized ground state geometries and partial charges were used to calculate the vertical excitation energies using the simplified time-dependent density functional theory (Bannwarth and Grimme, 2014; Runge and Gross, 1984) at the CAM-B3LYP/cc-pVTZ level of theory (Dunning, 1989; Yanai et al., 2004) using the Orca program. The excitation energies were also calculated using the RI-ADC(2) method (Hättig, 2005) with frozen core orbitals and the cc-pVTZ basis set in association with the corresponding auxiliary basis by utilizing the Turbomole 7.5.1 program package (Furche et al., 2014). The three-dimensional models were visualized using the molecular graphics program PyMOL 2.5.5.

## Data Availability

The cDNA sequences of Acropora tenuis opsins in this paper are available in GenBank: eight Cnidopsins (accession no. LC844924-LC844931), seven opsins in the ASO-II group (accession no. LC844932-LC844938), and one opsin in the ASO-I group (accession no. LC844939). The structural models of wild type Antho2a with a neutral or charged Glu292 and the Antho2a E292A mutant are available in Zenodo (10.5281/zenodo.15064942). All data needed to evaluate the conclusion in this paper are present in the paper and the source data files. The following datasets were generated: DeupiX SenS 2025Theoretical structural models of the anthozoan-specific opsin II Antho2a (wild type with Glu292 either neutral or negatively charged and the E292A mutant).Zenodo10.5281/zenodo.15064943 SakaiY SenS SugiharaT KakeyamaY IwasakiM SchertlerGFX DeupiX KoyanagiM TerakitaA 2025Acropora tenuis Cnidopsin1 CDSNCBI NucleotideLC844924 SakaiY SenS SugiharaT KakeyamaY IwasakiM SchertlerGFX DeupiX KoyanagiM TerakitaA 2025Acropora tenuis Cnidopsin2 CDSNCBI NucleotideLC844925 SakaiY SenS SugiharaT KakeyamaY IwasakiM SchertlerGFX DeupiX KoyanagiM TerakitaA 2025Acropora tenuis Cnidopsin3a CDSNCBI NucleotideLC844926 SakaiY SenS SugiharaT KakeyamaY IwasakiM SchertlerGFX DeupiX KoyanagiM TerakitaA 2025Acropora tenuis Cnidopsin3b CDSNCBI NucleotideLC844927 SakaiY SenS SugiharaT KakeyamaY IwasakiM SchertlerGFX DeupiX KoyanagiM TerakitaA 2025Acropora tenuis Cnidopsin4 CDSNCBI NucleotideLC844928 SakaiY SenS SugiharaT KakeyamaY IwasakiM SchertlerGFX DeupiX KoyanagiM TerakitaA 2025Acropora tenuis Cnidopsin5 CDSNCBI NucleotideLC844929 SakaiY SenS SugiharaT KakeyamaY IwasakiM SchertlerGFX DeupiX KoyanagiM TerakitaA 2025Acropora tenuis Cnidopsin6 CDSNCBI NucleotideLC844930 SakaiY SenS SugiharaT KakeyamaY IwasakiM SchertlerGFX DeupiX KoyanagiM TerakitaA 2025Acropora tenuis Cnidopsin7 CDSNCBI NucleotideLC844931 SakaiY SenS SugiharaT KakeyamaY IwasakiM SchertlerGFX DeupiX KoyanagiM TerakitaA 2025Acropora tenuis Antho2a CDSNCBI NucleotideLC844932 SakaiY SenS SugiharaT KakeyamaY IwasakiM SchertlerGFX DeupiX KoyanagiM TerakitaA 2025Acropora tenuis Antho2b CDSNCBI NucleotideLC844933 SakaiY SenS SugiharaT KakeyamaY IwasakiM SchertlerGFX DeupiX KoyanagiM TerakitaA 2025Acropora tenuis Antho2c CDSNCBI NucleotideLC844934 SakaiY SenS SugiharaT KakeyamaY IwasakiM SchertlerGFX DeupiX KoyanagiM TerakitaA 2025Acropora tenuis Antho2d CDSNCBI NucleotideLC844935 SakaiY SenS SugiharaT KakeyamaY IwasakiM SchertlerGFX DeupiX KoyanagiM TerakitaA 2025Acropora tenuis Antho2e CDSNCBI NucleotideLC844936 SakaiY SenS SugiharaT KakeyamaY IwasakiM SchertlerGFX DeupiX KoyanagiM TerakitaA 2025Acropora tenuis Antho2f CDSNCBI NucleotideLC844937 SakaiY SenS SugiharaT KakeyamaY IwasakiM SchertlerGFX DeupiX KoyanagiM TerakitaA 2025Acropora tenuis Antho2g CDSNCBI NucleotideLC844938 SakaiY SenS SugiharaT KakeyamaY IwasakiM SchertlerGFX DeupiX KoyanagiM TerakitaA 2025Acropora tenuis Antho1 CDSNCBI NucleotideLC844939

## Additional files

MDAR checklist

## References

[bib1] Agostini S, Suzuki Y, Higuchi T, Casareto BE, Yoshinaga K, Nakano Y, Fujimura H (2012). Biological and chemical characteristics of the coral gastric cavity. Coral Reefs.

[bib2] Al-Horani FA, Al-Moghrabi SM, de Beer D (2003). The mechanism of calcification and its relation to photosynthesis and respiration in the scleractinian coral Galaxea fascicularis. Marine Biology.

[bib3] Bannwarth C, Grimme S (2014). A simplified time-dependent density functional theory approach for electronic ultraviolet and circular dichroism spectra of very large molecules. Computational and Theoretical Chemistry.

[bib4] Barott KL, Barron ME, Tresguerres M (2017). Identification of a molecular pH sensor in coral. Proceedings. Biological Sciences.

[bib5] Becke AD (1988). Density-functional exchange-energy approximation with correct asymptotic behavior. Physical Review A.

[bib6] Besaw JE, Ou WL, Morizumi T, Eger BT, Sanchez Vasquez JD, Chu JHY, Harris A, Brown LS, Miller RJD, Ernst OP (2020). The crystal structures of a chloride-pumping microbial rhodopsin and its proton-pumping mutant illuminate proton transfer determinants. The Journal of Biological Chemistry.

[bib7] Besaw JE, Reichenwallner J, De Guzman P, Tucs A, Kuo A, Morizumi T, Tsuda K, Sljoka A, Miller RJD, Ernst OP (2022). Low pH structure of heliorhodopsin reveals chloride binding site and intramolecular signaling pathway. Scientific Reports.

[bib8] Blatz PE, Mohler JH, Navangul HV (1972). Anion-induced wavelength regulation of absorption maxima of Schiff bases of retinal. Biochemistry.

[bib9] Cai WJ, Ma Y, Hopkinson BM, Grottoli AG, Warner ME, Ding Q, Hu X, Yuan X, Schoepf V, Xu H, Han C, Melman TF, Hoadley KD, Pettay DT, Matsui Y, Baumann JH, Levas S, Ying Y, Wang Y (2016). Microelectrode characterization of coral daytime interior pH and carbonate chemistry. Nature Communications.

[bib10] Capella-Gutiérrez S, Silla-Martínez JM, Gabaldón T (2009). trimAl: a tool for automated alignment trimming in large-scale phylogenetic analyses. Bioinformatics.

[bib11] Case DA, Betz RM, Cerutti DS, Cheatham TE, Darden TA, Duke RE, Giese TJ, Gohlke H, Goetz AW, Homeyer N, Izadi S, Janowski P, Kaus J, Kovalenko A, Lee TS, LeGrand S, Li P, Lin C, Luchko T, Luo R, Madej B, Mermelstein D, Merz KM, Monard G, Nguyen H, Nguyen HT, Omelyan I, Onufriev A, Roe DR, Roitberg A, Sagui C, Simmerling CL, Botello-Smith WM, Swails J, Walker RC, Wang J, Wolf RM, Wu X, Xiaoand L, Kollman PA (2016). AMBER 2016.

[bib12] Case DA, Aktulga HM, Belfon K, Ben-Shalom IY, Berryman JT, Brozell SR, Carvahol FS, Cerutti DS, Cheatham III TE, Cisneros GA, Cruzeiro VWD, Darden TA, Forouzesh N, Ghazimirsaeed M, Giambaşu G, Giese T, Gilson MK, Gohlke H, Goetz AW, Harris J, Huang Z, Izadi S, Izmailov SA, Kasavajhala K, Kaymak MC, Kolossváry I, Kovalenko A, Kurtzman T, Lee TS, Li P, Li Z, Lin C, Liu J, Luchko T, Luo R, Machado M, Manathunga M, Merz KM, Miao Y, Mikhailovskii O, Monard G, Nguyen H, O’Hearn KA, Onufriev A, Pan F, Pantano S, Rahnamoun A, Roe DR, Roitberg A, Sagui C, Schott-Verdugo S, Shajan A, Shen J, Simmerling CL, Skrynnikov NRJ, Smith J, Swails J, Walker RC, Wang J, Wang J, Wu X, We Y, Xiong Y, Xue Y, York DM, Zhao C, Zhu Q, Kollman PA (2025).

[bib13] Church JR, Olsen JMH, Schapiro I (2021). The impact of retinal configuration on the protein-chromophore interactions in bistable jumping spider Rhodopsin-1. Molecules.

[bib14] Darriba D, Posada D, Kozlov AM, Stamatakis A, Morel B, Flouri T (2020). Model test-NG: a new and scalable tool for the selection of DNA and protein evolutionary models. Molecular Biology and Evolution.

[bib15] de la Fuente S, Fonteriz RI, de la Cruz PJ, Montero M, Alvarez J (2012). Mitochondrial free [Ca(2+)] dynamics measured with a novel low-Ca(2+) affinity aequorin probe. The Biochemical Journal.

[bib16] Dryja TP, Berson EL, Rao VR, Oprian DD (1993). Heterozygous missense mutation in the rhodopsin gene as a cause of congenital stationary night blindness. Nature Genetics.

[bib17] Dunning TH (1989). Gaussian basis sets for use in correlated molecular calculations. I. The atoms boron through neon and hydrogen. The Journal of Chemical Physics.

[bib18] Feuda R, Hamilton SC, Mclnerney JO, Pisani D (2012). Metazoan opsin evolution reveals a simple route to animal vision. PNAS.

[bib19] Foo SA, Liddell L, Grossman A, Caldeira K (2020). Photo-movement in the sea anemone Aiptasia influenced by light quality and symbiotic association. Coral Reefs.

[bib20] Furche F, Ahlrichs R, Hättig C, Klopper W, Sierka M, Weigend F (2014). Turbomole. WIREs Computational Molecular Science.

[bib21] Gerrard E, Mutt E, Nagata T, Koyanagi M, Flock T, Lesca E, Schertler GFX, Terakita A, Deupi X, Lucas RJ (2018). Convergent evolution of tertiary structure in rhodopsin visual proteins from vertebrates and box jellyfish. PNAS.

[bib22] Gornik SG, Bergheim BG, Morel B, Stamatakis A, Foulkes NS, Guse A (2021). Photoreceptor diversification accompanies the evolution of Anthozoa. Molecular Biology and Evolution.

[bib23] Grimme S, Ehrlich S, Goerigk L (2011). Effect of the damping function in dispersion corrected density functional theory. Journal of Computational Chemistry.

[bib24] Gruhl T, Weinert T, Rodrigues MJ, Milne CJ, Ortolani G, Nass K, Nango E, Sen S, Johnson PJM, Cirelli C, Furrer A, Mous S, Skopintsev P, James D, Dworkowski F, Båth P, Kekilli D, Ozerov D, Tanaka R, Glover H, Bacellar C, Brünle S, Casadei CM, Diethelm AD, Gashi D, Gotthard G, Guixà-González R, Joti Y, Kabanova V, Knopp G, Lesca E, Ma P, Martiel I, Mühle J, Owada S, Pamula F, Sarabi D, Tejero O, Tsai CJ, Varma N, Wach A, Boutet S, Tono K, Nogly P, Deupi X, Iwata S, Neutze R, Standfuss J, Schertler G, Panneels V (2023). Ultrafast structural changes direct the first molecular events of vision. Nature.

[bib25] Hättig C, Jensen HJÅ (2005). Response Theory and Molecular Properties (A Tribute to Jan Linderberg and Poul Jørgensen), Advances in Quantum Chemistry.

[bib26] Hering L, Mayer G (2014). Analysis of the opsin repertoire in the tardigrade Hypsibius dujardini provides insights into the evolution of opsin genes in panarthropoda. Genome Biology and Evolution.

[bib27] Hofmann KP, Scheerer P, Hildebrand PW, Choe HW, Park JH, Heck M, Ernst OP (2009). A G protein-coupled receptor at work: the rhodopsin model. Trends in Biochemical Sciences.

[bib28] Hosaka T, Yoshizawa S, Nakajima Y, Ohsawa N, Hato M, DeLong EF, Kogure K, Yokoyama S, Kimura-Someya T, Iwasaki W, Shirouzu M (2016). structural mechanism for light-driven transport by a new type of chloride ion pump, nonlabens marinus Rhodopsin-3. The Journal of Biological Chemistry.

[bib29] Jin S, Cornwall MC, Oprian DD (2003). Opsin activation as a cause of congenital night blindness. Nature Neuroscience.

[bib30] Jorgensen WL, Chandrasekhar J, Madura JD, Impey RW, Klein ML (1983). Comparison of simple potential functions for simulating liquid water. The Journal of Chemical Physics.

[bib31] Jumper J, Evans R, Pritzel A, Green T, Figurnov M, Ronneberger O, Tunyasuvunakool K, Bates R, Žídek A, Potapenko A, Bridgland A, Meyer C, Kohl SAA, Ballard AJ, Cowie A, Romera-Paredes B, Nikolov S, Jain R, Adler J, Back T, Petersen S, Reiman D, Clancy E, Zielinski M, Steinegger M, Pacholska M, Berghammer T, Bodenstein S, Silver D, Vinyals O, Senior AW, Kavukcuoglu K, Kohli P, Hassabis D (2021). Highly accurate protein structure prediction with AlphaFold. Nature.

[bib32] Katoh K, Standley DM (2013). MAFFT multiple sequence alignment software version 7: improvements in performance and usability. Molecular Biology and Evolution.

[bib33] Kayada S, Hisatomi O, Tokunaga F (1995). Cloning and expression of frog rhodopsin cDNA. Comp Biochem Physiol B Biochem Mol Biol.

[bib34] Kishimoto M, Gornik SG, Foulkes NS, Guse A (2023). Negative phototaxis in the photosymbiotic sea anemone Aiptasia as a potential strategy to protect symbionts from photodamage. Scientific Reports.

[bib35] Kolbe M, Besir H, Essen LO, Oesterhelt D (2000). Structure of the light-driven chloride pump halorhodopsin at 1.8 A resolution. Science.

[bib36] Kouyama T, Kanada S, Takeguchi Y, Narusawa A, Murakami M, Ihara K (2010). Crystal structure of the light-driven chloride pump halorhodopsin from Natronomonas pharaonis. Journal of Molecular Biology.

[bib37] Koyanagi M, Takano K, Tsukamoto H, Ohtsu K, Tokunaga F, Terakita A (2008). Jellyfish vision starts with cAMP signaling mediated by opsin-G(s) cascade. PNAS.

[bib38] Koyanagi M, Terakita A (2014). Diversity of animal opsin-based pigments and their optogenetic potential. Biochimica et Biophysica Acta.

[bib39] Koyanagi M, Shen B, Nagata T, Sun L, Wada S, Kamimura S, Kage-Nakadai E, Terakita A (2022). High-performance optical control of GPCR signaling by bistable animal opsins MosOpn3 and LamPP in a molecular property-dependent manner. PNAS.

[bib40] Kozlov AM, Darriba D, Flouri T, Morel B, Stamatakis A (2019). RAxML-NG: a fast, scalable and user-friendly tool for maximum likelihood phylogenetic inference. Bioinformatics.

[bib41] Kozmik Z, Ruzickova J, Jonasova K, Matsumoto Y, Vopalensky P, Kozmikova I, Strnad H, Kawamura S, Piatigorsky J, Paces V, Vlcek C (2008). Assembly of the cnidarian camera-type eye from vertebrate-like components. PNAS.

[bib42] Levy S, Elek A, Grau-Bové X, Menéndez-Bravo S, Iglesias M, Tanay A, Mass T, Sebé-Pedrós A (2021). A stony coral cell atlas illuminates the molecular and cellular basis of coral symbiosis, calcification, and immunity. Cell.

[bib43] Maier JA, Martinez C, Kasavajhala K, Wickstrom L, Hauser KE, Simmerling C (2015). ff14SB: improving the accuracy of protein side chain and backbone parameters from ff99SB. Journal of Chemical Theory and Computation.

[bib44] Mason BM, Koyanagi M, Sugihara T, Iwasaki M, Slepak V, Miller DJ, Sakai Y, Terakita A (2023). Multiple opsins in a reef-building coral, Acropora millepora. Scientific Reports.

[bib45] Mayol E, García-Recio A, Tiemann JKS, Hildebrand PW, Guixà-González R, Olivella M, Cordomí A (2020). HomolWat: a web server tool to incorporate “homologous” water molecules into GPCR structures. Nucleic Acids Research.

[bib46] Metz S, Kästner J, Sokol AA, Keal TW, Sherwood P (2014). ChemShell—a modular software package for QM/MM simulations. Wiley Interdisciplinary Reviews. Computational Molecular Science.

[bib47] Mizuno M, Nakajima A, Kandori H, Mizutani Y (2018). Structural Evolution of a Retinal Chromophore in the Photocycle of Halorhodopsin from Natronobacterium pharaonis. The Journal of Physical Chemistry. A.

[bib48] Mous S, Gotthard G, Ehrenberg D, Sen S, Weinert T, Johnson PJM, James D, Nass K, Furrer A, Kekilli D, Ma P, Brünle S, Casadei CM, Martiel I, Dworkowski F, Gashi D, Skopintsev P, Wranik M, Knopp G, Panepucci E, Panneels V, Cirelli C, Ozerov D, Schertler GFX, Wang M, Milne C, Standfuss J, Schapiro I, Heberle J, Nogly P (2022). Dynamics and mechanism of a light-driven chloride pump. Science.

[bib49] Mroginski M-A, Adam S, Amoyal GS, Barnoy A, Bondar A-N, Borin VA, Church JR, Domratcheva T, Ensing B, Fanelli F, Ferré N, Filiba O, Pedraza-González L, González R, González-Espinoza CE, Kar RK, Kemmler L, Kim SS, Kongsted J, Krylov AI, Lahav Y, Lazaratos M, NasserEddin Q, Navizet I, Nemukhin A, Olivucci M, Olsen JMH, Pérez de Alba Ortíz A, Pieri E, Rao AG, Rhee YM, Ricardi N, Sen S, Solov’yov IA, De Vico L, Wesolowski TA, Wiebeler C, Yang X, Schapiro I (2021). Frontiers in multiscale modeling of photoreceptor proteins. Photochemistry and Photobiology.

[bib50] Nagata T, Koyanagi M, Tsukamoto H, Mutt E, Schertler GFX, Deupi X, Terakita A (2019). The counterion-retinylidene Schiff base interaction of an invertebrate rhodopsin rearranges upon light activation. Communications Biology.

[bib51] Nathans J (1990). Determinants of visual pigment absorbance: identification of the retinylidene Schiff’s base counterion in bovine rhodopsin. Biochemistry.

[bib52] Neese F (2022). Software update: the ORCA program system—version 5.0. Wiley Interdisciplinary Reviews. Computational Molecular Science.

[bib53] Obayashi K, Zou R, Kawaguchi T, Mori T, Tsukamoto H (2025). Molecular basis underlying the specificity of an antagonist AA92593 for mammalian melanopsins. The Journal of Biological Chemistry.

[bib54] Olsson MHM, Søndergaard CR, Rostkowski M, Jensen JH (2011). PROPKA3: Consistent treatment of internal and surface residues in empirical pKa predictions. Journal of Chemical Theory and Computation.

[bib55] Picciani N, Kerlin JR, Sierra NC, Swafford AJM, Ramirez MD, Cannon JT, Daly M, Oakley TH (2018). Prolific origination of eyes in cnidaria with co-option of non-visual opsins. SSRN Electronic Journal.

[bib56] Pitt GA, Collins FD, Morton RA, Stok P (1955). Studies on rhodopsin. VIII. Retinylidenemethylamine, an indicator yellow analogue. The Biochemical Journal.

[bib57] Ramirez MD, Pairett AN, Pankey MS, Serb JM, Speiser DI, Swafford AJ, Oakley TH (2016). The last common ancestor of most bilaterian animals possessed at least nine opsins. Genome Biology and Evolution.

[bib58] Ridge KD, Abdulaev NG (2000). Folding and assembly of rhodopsin from expressed fragments. Methods in Enzymology.

[bib59] Runge E, Gross EKU (1984). Density-functional theory for time-dependent systems. Physical Review Letters.

[bib60] Ryckaert JP, Ciccotti G, Berendsen HJC (1977). Numerical integration of the cartesian equations of motion of a system with constraints: molecular dynamics of n-alkanes. Journal of Computational Physics.

[bib61] Sakai K, Ikeuchi H, Fujiyabu C, Imamoto Y, Yamashita T (2022). Convergent evolutionary counterion displacement of bilaterian opsins in ciliary cells. Cellular and Molecular Life Sciences.

[bib62] Sakmar TP, Franke RR, Khorana HG (1989). Glutamic acid-113 serves as the retinylidene Schiff base counterion in bovine rhodopsin. PNAS.

[bib63] Sakmar TP, Franke RR, Khorana HG (1991). The role of the retinylidene Schiff base counterion in rhodopsin in determining wavelength absorbance and Schiff base pKa. PNAS.

[bib64] Scharf B, Engelhard M (1994). Blue halorhodopsin from Natronobacterium pharaonis: wavelength regulation by anions. Biochemistry.

[bib65] Senn HM, Thiel W (2009). QM/MM methods for biomolecular systems. Angewandte Chemie.

[bib66] Sherwood P, de Vries AH, Guest MF, Schreckenbach G, Catlow CRA, French SA, Sokol AA, Bromley ST, Thiel W, Turner AJ, Billeter S, Terstegen F, Thiel S, Kendrick J, Rogers SC, Casci J, Watson M, King F, Karlsen E, Sjøvoll M, Fahmi A, Schäfer A, Lennartz C (2003). QUASI: A general purpose implementation of the QM/MM approach and its application to problems in catalysis. Journal of Molecular Structure.

[bib67] Shichida Y, Kato T, Sasayama S, Fukada Y, Yoshizawa T (1990). Effects of chloride on chicken iodopsin and the chromophore transfer reactions from iodopsin to scotopsin and B-photopsin. Biochemistry.

[bib68] Shihoya W, Inoue K, Singh M, Konno M, Hososhima S, Yamashita K, Ikeda K, Higuchi A, Izume T, Okazaki S, Hashimoto M, Mizutori R, Tomida S, Yamauchi Y, Abe-Yoshizumi R, Katayama K, Tsunoda SP, Shibata M, Furutani Y, Pushkarev A, Béjà O, Uchihashi T, Kandori H, Nureki O (2019). Crystal structure of heliorhodopsin. Nature.

[bib69] Shinzato C, Khalturin K, Inoue J, Zayasu Y, Kanda M, Kawamitsu M, Yoshioka Y, Yamashita H, Suzuki G, Satoh N (2021). Eighteen coral genomes reveal the evolutionary origin of *Acropora* strategies to accommodate environmental changes. Molecular Biology and Evolution.

[bib70] Sinha A, Jones Brunette AM, Fay JF, Schafer CT, Farrens DL (2014). Rhodopsin TM6 can interact with two separate and distinct sites on arrestin: evidence for structural plasticity and multiple docking modes in arrestin-rhodopsin binding. Biochemistry.

[bib71] Søndergaard CR, Olsson MHM, Rostkowski M, Jensen JH (2011). Improved treatment of ligands and coupling effects in empirical calculation and rationalization of pKa values. Journal of Chemical Theory and Computation.

[bib72] Steinberg G, Ottolenghi M, Sheves M (1993). pKa of the protonated Schiff base of bovine rhodopsin: A study with artificial pigments. Biophysical Journal.

[bib73] Suga H, Schmid V, Gehring WJ (2008). Evolution and functional diversity of jellyfish opsins. Current Biology.

[bib74] Terakita A, Hara R, Hara T (1989). Retinal-binding protein as a shuttle for retinal in the rhodopsin-retinochrome system of the squid visual cells. Vision Research.

[bib75] Terakita A, Yamashita T, Shichida Y (2000). Highly conserved glutamic acid in the extracellular IV-V loop in rhodopsins acts as the counterion in retinochrome, a member of the rhodopsin family. PNAS.

[bib76] Terakita A, Koyanagi M, Tsukamoto H, Yamashita T, Miyata T, Shichida Y (2004). Counterion displacement in the molecular evolution of the rhodopsin family. Nature Structural & Molecular Biology.

[bib77] Terakita A (2005). The opsins. Genome Biology.

[bib78] Terakita A, Kawano‐Yamashita E, Koyanagi M (2012). Evolution and diversity of opsins. Wiley Interdisciplinary Reviews.

[bib79] Tsutsui K, Shichida Y (2010). Photosensitivities of rhodopsin mutants with a displaced counterion. Biochemistry.

[bib80] Venn AA, Tambutté E, Lotto S, Zoccola D, Allemand D, Tambutté S (2009). Imaging intracellular pH in a reef coral and symbiotic anemone. PNAS.

[bib81] Voolstra CR, Miller DJ, Ragan MA, Hoffmann AA, Hoegh-Guldberg O, Bourne DG, Ball EE, Ying H, Forêt S, Takahashi S, Weynberg KD, Oppen MJH, Morrow K, Chan CX, Rosic N, Leggat W, Sprungala S, Imelfort M, Tyson GW, Kassahn KS, Lundgren PB, Beeden RJ, Ravasi T, Berumen ML, Abal E, Fyffe T (2015). The ReFuGe 2020 Consortium—using “omics” approaches to explore the adaptability and resilience of coral holobionts to environmental change. Frontiers in Marine Science.

[bib82] Weigend F (2008). Hartree-Fock exchange fitting basis sets for H to Rn. Journal of Computational Chemistry.

[bib83] Yanai T, Tew DP, Handy NC (2004). A new hybrid exchange–correlation functional using the Coulomb-attenuating method (CAM-B3LYP). Chemical Physics Letters.

[bib84] Zhukovsky EA, Oprian DD (1989). Effect of carboxylic acid side chains on the absorption maximum of visual pigments. Science.

